# Safety and Efficacy of Treatment with/without Ramucirumab in Advanced or Metastatic Cancer: A Meta-Analysis of 11 Global, Double-Blind, Phase 3 Randomized Controlled Trials

**DOI:** 10.1155/2022/2476469

**Published:** 2022-11-21

**Authors:** Lingxue Tang, Tong Wang, Qianqian Zhang, Sheng Yu, Wen Li, Senbang Yao, Huaidong Cheng

**Affiliations:** ^1^Department of Oncology, The Second Affiliated Hospital of Anhui Medical University, Hefei, Anhui, China; ^2^Department of General Medicine, The Second Affiliated Hospital of Anhui Medical University, Hefei, Anhui, China; ^3^Department of Oncology, Shenzhen Hospital of Southern Medical University, Shenzhen, Guangdong, China

## Abstract

Ramucirumab, as a vascular endothelial growth factor receptor-2 inhibitor, was first approved in 2014 for treated advanced or metastatic gastric/gastroesophageal junction (GEJ) adenocarcinoma. This study deeply analyzed the efficacy and safety of advanced or metastatic cancer treated with ramucirumab, which included 11 global, double-blind, phase 3 randomized controlled trials with a total of 7410 patients. Subgroup analysis based on different cancer types showed that standard regimens plus ramucirumab significantly increased progression-free survival and overall survival compared with placebo groups in patients with advanced non-small-cell lung cancer (NSCLC), hepatocellular carcinoma, gastric cancer, or GEJ adenocarcinoma. Although a higher proportion of patients achieved overall response and disease control than those treated with placebo, the overall response was not statistically significant between the two groups in advanced NSCLC. Grade 3 or worse treatment-emergent adverse events (TEAEs) that occurred in at least 5% of patients were neutropenia (30.5% in the ramucirumab group vs. 23.5% in the placebo group), leucopenia (14.8% vs. 9.2%), weight decreased (14.2% vs. 8.0%), myalgia (11.7% vs. 7.7%), fatigue (10.9% vs. 7.7%), hypertension (9.2% vs. 2.3%), and anaemia (6.2% vs. 7.7%). In the TEAEs of special interest, the ramucirumab group had a significantly higher incidence of bleeding (mainly grade 1-2 epistaxis and gastrointestinal bleeding), hypertension, proteinuria, liver injury/failure (grade 1-2), venous thromboembolism (grade 1-2), and gastrointestinal perforation (grade ≧3) than the control group.

## 1. Introduction

Cancer is a serious global public health problem and is expected to become the leading cause of death in countries around the world in the 21st century, which may bring great harm to human health [1]. In 2021, there was an estimation of 1,898,160 new cancer cases and 608,570 cancer-related deaths in the United States [2]. In the special period of the COVID-19 global pandemic, the delay in diagnosis and treatment of early cancer may further increase the incidence and mortality of advanced cancer due to the scarcity of medical resources. This phenomenon inevitably leads to a heavier economic burden on families and society. Traditional treatments for cancer include surgery, chemotherapy, and radiation. However, due to the untimely early diagnosis, most patients lose the opportunity for surgical treatment. Chemotherapy or radiotherapy also primer its acquired drug resistance and serious side effects, forcing people to seek other treatments. Therefore, the combination of targeted therapies, immunotherapy, and gene therapy has developed rapidly in recent years [3].

The formation of blood vessels can provide oxygen and nutrients to tissues and play an important role in the growth, invasion, and metastasis of tumor cells [4]. This hypothesis was first proposed by Folkman in 1971, who believed that the continuous growth of solid tumors larger than 1-2 mm^3^ requires new blood vessels to promote, and gave some evidence to support this view in subsequent studies [5, 6]. In the past 50 years, a large number of studies have reported the effects of a variety of regulatory factors on tumor angiogenesis, such as vascular endothelial growth factor (VEGF), transforming growth factor, and hypoxia-inducible factor [7–10]. VEGF is one of the targets of anticancer drugs. Ligands VEGF-A, -C, and -D can specifically bind to receptor VEGFR-2, increase angiogenesis and vascular permeability, and provide sufficient oxygen and nutrients to cancer cells [11]. Therefore, limiting the binding of VEGF ligands and receptors may delay the progression of cancer. An abnormal expression of VEGF has been found in various types of cancer and is associated with poor prognosis. For example, digestive cancers included esophageal cancer [12], gastric cancer [13], colorectal cancer [14], intrahepatic cholangiocarcinoma [15], and pancreatic cancer [12], as well as nondigestive cancers, included lung cancer [16], breast [17], cervical cancer [18], ovarian cancer [19], osteosarcoma [20], prostate cancer [21], and renal cancer [22]. VEGF is therefore a promising therapeutic target in advanced or metastatic cancers. The VEGFR-2 inhibitor ramucirumab, a fully human immunoglobulin G1 monoclonal antibody, was first approved in the United States in 2014 for the treatment of advanced or metastatic gastric/gastroesophageal junction (GEJ) adenocarcinoma [23–25]. Today, results from 11 worldwide, phase 3, double-blind randomized controlled trials (RCTs) investigating the efficacy and safety of RAM in different types of cancer have been published, and a number of Phase 1/2 RCTs is also underway. Based on the data reported in these 11 worldwide, phase 3 RCTs, the present study's purpose is to investigate whether adding ramucirumab to standard regimens could improve the treatment outcomes in advanced or metastatic cancer, including overall survival (OS), progression-free survival (PFS), objective response rate (ORR), disease control rate (DCR), treatment-emergent adverse events (TEAEs), or of special interest, and to evaluate the efficacy and safety of combination treatment.

## 2. Methods

The present study was conducted based on the PRISMA guidelines (registration number: CRD42022324190; https://www.crd.york.ac.uk/prospero/).

### 2.1. Search Strategy

The literature search included standard regimens with/without ramucirumab treatment for advanced or metastatic cancer by PubMed, Web of Science, and the Cochrane Library databases, as well as three international conferences including the American Society of Clinical Oncology and European Cancer Conference without language restrictions. The deadline was on January 31, 2022.  Table S1 shows the search strategy of the PubMed database; other databases' search strategy is similar to this. Besides, we manually searched the references of relevant articles to find other articles that meet the research conditions.

### 2.2. Inclusion and Exclusion Criteria

The inclusion criteria were as follows: (1) patients who were diagnosed with advanced or metastatic cancer cytologically or histologically, (2) published phase 3 RCTs, (3) the experimental group was treated by standard regimens (chemotherapy, radiotherapy, targeted therapy, and best supportive care) plus ramucirumab, and the control group was standard regimens plus placebo, and (4) at least one outcome was reported (PFS, OS, ORR, DCR, TEAEs, or of special interest). Articles or abstracts that do not meet any of the abovementioned criteria were excluded.

### 2.3. Data Extraction

The titles and abstracts of published articles were first selected according to the keywords. After preliminary evaluation, the full texts that meet the collection requirements were downloaded for in-depth reading. Second, the detailed data of qualified studies, including first author, title, year of publication, treatment measures, sample size, disease stage, and primary and secondary outcomes, were independently extracted into two spreadsheets by two authors, and the authenticity of these data was carefully checked by the other two authors to avoid possible evaluation bias caused by researchers. If a study has more than one publication, the authors aggregated the latest results for data analysis. The authors did not extract data for all of the adverse events mentioned in the study. Instead, only treatment-emergent adverse events reported in more than two studies were analyzed. Any differences of opinion were resolved by discussion with the principal investigator or arbitration by a third examiner.

### 2.4. Quality Evaluation

The risk of bias for each article was assessed using the Cochrane Collaboration tool, and the following evaluation domains were assessed accordingly: random sequence generation, allocation concealment, blinding of participants and personnel, blinding of outcome assessment, incomplete outcome data, selective reporting, and other biases [26]. Review Manager (version 5.4), a statistical software provided by the Cochrane Collaboration, was used to draw the “Risk of bias graph” (Figure S1) and “Risk of bias summary” (Figure S2).

### 2.5. Statistical Analysis

Data analysis and figures production were completed by the Review Manager. PFS and OS were analyzed by the hazard ratio (HR) and 95% confidence interval (CI). ORR, DCR, and adverse events were analyzed by the odds ratio (OR) and 95%CI, *p*  < 0.05 means a statistical significance. The heterogeneity of studies meeting inclusion criteria were assessed by I-square and chi-square tests. When *I*^2^ > 50% or *p*≦0.1, the random-effect model was adopted; if not, the fixed-effect model was used.

## 3. Results

**Figure 1:**
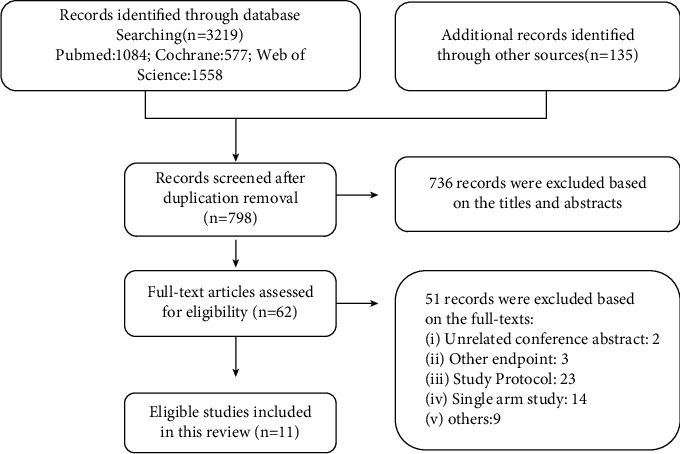
Flowchart of articles included in the meta-analysis. Articles that meet the inclusion requirements were screened according to PRISMA guidelines, and PICO principles were followed (population, interventions, comparisons, and outcomes).

This study retrieved a total of 3354 articles from the database, of which 736 did not meet the inclusion criteria and 2560 duplicated studies were excluded. After in-depth reading of the full text, 11 RCTs were finally included in this meta-analysis (Figure 1). The main characteristics and dosage administered of each RCTs are shown in Table 1, including RAINBOW-Asia [27], RELAY [28], REACH-2 [29], RANGE [30], RAINFALL [31], RAINBOW [32], RAISE [33], REVEL [34], REACH [35], REGARD [36], and ROSE/TRIO-012 [37]. There were 7 RCTs compared the chemotherapy plus ramucirumab with chemotherapy plus placebo, 3 RCTs compared best supportive care (BSC) plus ramucirumab after chemotherapy with BSC plus placebo after chemotherapy, and 1 RCT compared EGFR-TKI plus ramucirumab with EGFR-TKI plus placebo. The total population was 7410, with 4078 (55%) in the ramucirumab group and 3332 (45%) in the control groups. The remaining characteristics are shown in Tables 2 and S2.

### 3.1. PFS and OS

**Figure 2:**
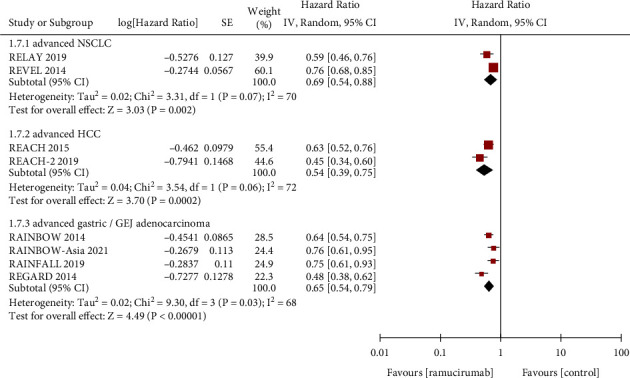
Forest plots showing the hazard ratio for progression-free survival.

**Figure 3:**
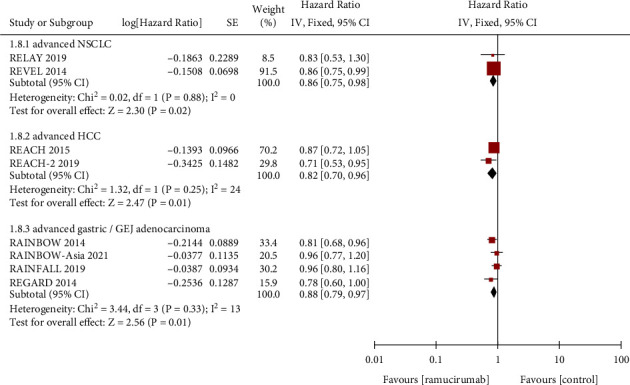
Forest plots showing the hazard ratio for overall survival.

PFS is defined as the time of disease progression or death from any cause since randomization, and OS is defined as the time from randomization to the date of death from any cause. Meta-analysis of PFS (Figure 2) and OS (Figure 3) were performed according to different cancer types.

2 of the 11 RCTs were reported PFS and OS in a total of 1702 patients with advanced non-small-cell lung cancer (NSCLC). The pooled HR was 0.69 (95%CI: 0.54–0.88, *p*=0.002) in PFS and 0.86 (95%CI: 0.75–0.98, *p*=0.02) in OS. 2 RCTs were reported in 857 patients with advanced hepatocellular carcinoma (HCC). The pooled HR was 0.54 (95%CI: 0.39–0.75, *p*=0.0002) in PFS and 0.82 (95%CI: 0.70–0.96, *p*=0.01) in OS. Moreover, a total of 4 RCTs were reported in 2105 patients with advanced gastric cancer or GEJ adenocarcinoma. The pooled HR was 0.65 (95%CI: 0.54–0.79, *p* < 0.00001) in PFS and 0.88 (95%CI: 0.79–0.97, *p*=0.01) in OS. In addition, the remaining 3 RCTs were reported on advanced colorectal carcinoma, breast cancer, and urothelial carcinoma respectively, so we could not conduct a meta-analysis on them. In general, the abovementioned results indicated that the standard regimen combined with ramucirumab could achieve more beneficial PFS and OS than the standard regimen plus placebo in patients with advanced NSCLC, HCC, gastric cancer, or GEJ adenocarcinoma.

### 3.2. ORR and DCR

**Figure 4:**
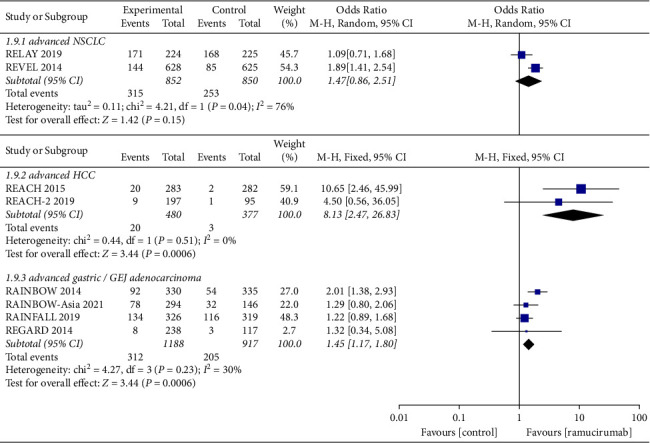
Forest plots showing the odds ratio for objective response rate.

**Figure 5:**
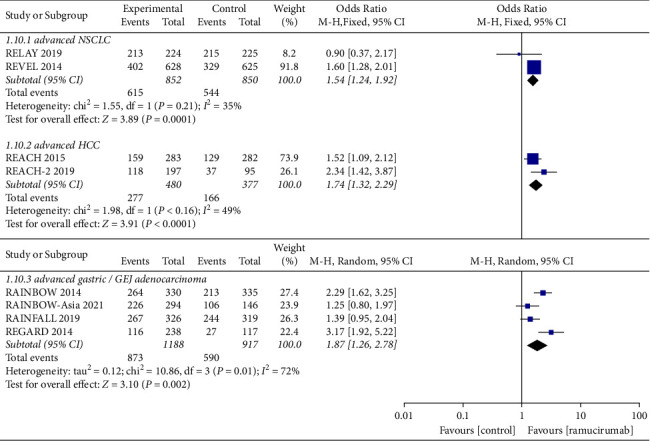
Forest plots showing the odds ratio for disease control rate.

ORR is defined as complete response rate plus partial response rate. DCR is defined as complete response rate plus partial response rate and disease stability rate. Similarly, a meta-analysis of ORR (Figure 4) and DCR (Figure 5) was also conducted according to different cancer types.

In advanced NSCLC, the ORR reported by the ramucirumab group and control group were 37.0% and 29.8%, and the DCR in the two groups were 72.2% and 64.0%. The pooled OR was 1.47 (95%CI: 0.86–2.51, *p*=0.15) in ORR and 1.59 (95%CI: 1.24–1.92, *p*=0.0001) in DCR. In advanced HCC, the ORR values reported by the ramucirumab group and control group were 6.0% and 0.8%, and the DCR values in two groups were 57.7% and 44.0%, respectively. The pooled OR was 8.13 (95%CI: 2.47–26.83, *p*=0.0006) in ORR and 1.74 (95%CI: 1.32–2.29, *p* < 0.0001) in DCR. In advanced gastric cancer or GEJ adenocarcinoma, the ORR values reported by the ramucirumab group and control group were 26.3% and 22.4%, and the DCR in the two groups were 73.5% and 64.3%, respectively. The pooled OR was 1.45 (95%CI: 1.17–1.80, *p*=0.0006) in ORR and 1.87 (95%CI: 1.26–2.78, *p*=0.002) in DCR, respectively.

In conclusion, compared with the control group, the ramucirumab group brought better ORR and DCR to patients with advanced HCC, gastric cancer, or GEJ adenocarcinoma. It should be noted that the proportion of patients with advanced HCC who have achieved disease control was very low (6.0% vs. 0.8%). For patients with advanced NSCLC, although the DCR of the ramucirumab group was better than the control group, the ORR between the two groups was not statistically significant.

### 3.3. TEAEs of Special Interest

The merged data of grade 1-2 and grade≧3 TEAEs of special interest were analyzed, respectively (Table 3). The number of events in each RCT and the raw data for the meta-analysis were provided in the supplementary material as Table S3.

#### 3.3.1. Bleeding/Haemorrhage

10 RCTs reported the risk of bleeding/haemorrhage events, including 1304 (34.5%) patients in the ramucirumab group and 503 (16.6%) in the control group with grade 1-2 events, 107 (2.8%) in the ramucirumab group and 87 (2.9%) in the control group with grade≧3. Although the results showed a higher incidence of bleeding/haemorrhage events in the ramucirumab group at grade 1-2 (OR: 2.72, 95% CI: 2.31–3.09, *p*  < 0.00001), there was no statistical significance between the two groups at grade≧3 (OR: 1.28, 95%CI: 0.82–1.99, *p*=0.27). Since pulmonary haemorrhage is a problem of NSCLC receiving antiangiogenic drugs, a separate meta-analysis of two studies (REVEL and RELAY) in advanced NSCLC was performed, and the results showed there were no statistically significant differences between the two groups in grade 1-2 (OR: 2.04, 95% CI: 0.45–9.33, *p*=0.36) or grade≧3 (OR: 0.88, 95% CI: 0.35–2.24, *p*=0.79) pulmonary haemorrhage events.

#### 3.3.2. Hypertension

Hypertension was defined as newly diagnosed arterial hypertension during treatment or a previously diagnosed hypertension that worsened during treatment. Hypertension (hypertension and increased blood pressure) data were provided in 10 RCTs, included 600 (15.9%) patients in the ramucirumab group and 190 (6.3%) in the control group with grade 1-2 events, 379 (10.0%) in the ramucirumab group and 88 (2.9%) in the control group with grade≧3. The results of different analysis between two groups in the occurrence of grade 1-2 and grade≧3 events were (OR: 2.57, 95%CI: 2.16–3.095, *p* < 0.00001) and (OR: 3.86, 95%CI: 3.04–4.89, *p* < 0.00001), respectively. In general, the incidence of hypertension in the ramucirumab group was higher than in the control group, regardless of grade 1-2 or grade≧3.

#### 3.3.3. Proteinuria

There were 486 (13.0%) patients in the ramucirumab group and 153 (5.0%) in the control group with grade 1–2 events, 55 (1.5%) in the ramucirumab group, and 4 (0.1%) in the control group with grade≧3. Moreover, the meta-analysis showed that the incidence of proteinuria in the ramucirumab group was higher than in the control group, regardless of grade 1–2 (OR: 2.94, 95%CI: 2.42–3.57, *p* < 0.00001) or gra de≧3 (OR: 6.46, 95%CI: 3.03–13.78, *p* < 0.00001).

#### 3.3.4. Liver Injury/Failure

5 RCTs reported *Liver Injury/Failure*, included 361 (22.2%) patients in the ramucirumab group and 170 (12.4%) in the control group with grade 1-2 events, 155 (9.6%) in the ramucirumab group and 125 (9.1%) in the control group with grade≧3. The incidence of liver injury/failure in the ramucirumab group was higher than the control group at grade 1-2 (OR: 1.73, 95%CI: 1.40–2.15, *p* < 0.00001), but there was not statistically significant at grade≧3 (OR: 1.01, 95%CI: 0.78–1.31, *p*=0.92).

#### 3.3.5. Epistaxis

Epistaxis is a type of bleeding/haemorrhage, and it was analyzed separately in 4 RCTs, included 393 (25.0%) patients in the ramucirumab group and 149 (10.2%) in the control group with grade 1-2 events, 3 (0.2%) in the ramucirumab group and 1 (0.0%) in the control group with grade≧3. The incidence of epistaxis in the ramucirumab group was higher than the control group at grade 1–2 (OR: 3.19, 95%CI: 2.59–3.92, *p* < 0.00001], but there was no statistically significant difference at gra de≧3 (OR: 1.77, 95%CI: 0.26–12.18, *p*=0.56).

#### 3.3.6. Others

Congestive heart failure (CHF), infusion-related reaction (IRR), venous thromboembolic (VTE), arterial thromboembolic (ATE), gastrointestinal (GI) perforation, GI haemorrhage, renal failure, fistula, healing complication, and pulmonary hemorrhage were adverse events with low probability (<5%). There was no significant difference between the two groups except for the higher incidence of grade 1-2 VTE events (OR: 0.68, 95%CI: 0.53–0.89, *p*=0.004), GI haemorrhage events (OR: 1.88, 95%CI: 1.44–2.46, *p* < 0.00001), and gra de≧3 GI perforation events (OR: 3.24, 95%CI: 1.60–6.57, *p*=0.001) in the ramucirumab group.

### 3.4. TEAEs

The merged data of grade 1-2 and gra de≧3 TEAEs were analyzed, respectively (Table 4). The number of events in each RCT and the raw data for the meta-analysis were provided in the supplementary material as Table S4. In the total incidence of TEAEs, fatigue (55.2%) and neutropenia (52.3%) were the most common, followed by diarrhoea (33.5%), nausea (33.0%), alopecia (29.1%), stomatitis (28.3%), decreased appetite (27.8%), leucopenia (26.7%), anaemia (25.1%), and the incidence rate of other TEAEs was 10%–25%. In TEAEs, the incidence of grade 1-2 and grade≧3 fatigue, hypertension, decreased appetite, stomatitis, neutropenia, and thrombocytopenia in the ramucirumab group were higher than in the control group. The incidence of grade 1-2 nausea, epistaxis, peripheral oedema, pyrexia, proteinuria, cough, hypoalbuminaemia, ascites, headache, leucopenia events, or grade≧3 neuropathy, myalgia events were also higher in the ramucirumab group than in the control group. However, abdominal pain, alopecia, diarrhoea, dyspnoea, vomiting, back pain, weight decreased, and rash were not statistically different between the two groups. It is worth noting that the incidence of grade≧3 constipation events and all grade anaemia events were higher in the control group.

### 3.5. Subgroup Analysis Based on Different Treatment Regimens

Subgroup meta-analyses of TEAEs of special interest based on different treatment regimens also have been performed (Table 5). The incidence of all grades of hypertensive events, grade 1-2 proteinuria, and bleeding/haemorrhage events was higher in the ramucirumab group than the control group, regardless of what treatment regimens were combined. Besides, the addition of ramucirumab also increased the incidence of grade 1-2 liver injury/failure events and grade≧3 proteinuria events in BSC. However, the incidence of grade≧3 VTE events was higher in the control group. Furthermore, the addition of ramucirumab increased the incidence of grade 1-2 liver injury/failure events in PTX, and all grades of VTE events in DOC.

## 4. Discussion

To our knowledge, ramucirumab plus BSC, targeted therapy, or chemotherapy significantly increased OS compared with placebo groups in patients with advanced NSCLC, HCC, gastric cancer, or GEJ adenocarcinoma. These patients treated with ramucirumab also had significantly longer PFS. Although a higher proportion of patients achieved overall response and disease control than those treated with a placebo, the ORR was not statistically significant between the two groups in advanced NSCLC.

In the TEAEs that were statistically different between the two groups, fatigue, diarrhoea (grade 3), nausea (grade 1–2), decreased appetite, and stomatitis were some of the most frequently reported nonhaematological events. Neutropenia, leucopenia (grade 1–2), and thrombocytopenia were the most frequently reported haematological TEAEs. Moreover, the incidence of TEAEs reported above was higher in the ramucirumab group. In addition, the TEAEs of special interest such as bleeding/haemorrhage (mainly grade 1–2 epistaxis and gastrointestinal haemorrhage), hypertension, liver injury/failure (grade 1–2), proteinuria, VTE (grade 1–2), and GI perforation (grade 3) were also more common in the ramucirumab group, as expected, consistent with the incidence of adverse events associated with most antiangiogenic therapies (38–40). Most of grade 4 or 5 TEAEs of special interest occurred with a very low incidence in both groups. There were only 0.07% of patients (3/4040) reported grade 4 hypertensive crisis, and 0.03% of patients (1/3782) reported grade 4 proteinuria in the ramucirumab group (0% in control), with no grade 5 events. Grade 4 and grade 5 GI perforation events were 0.36% (10/2783) and 0.22% (6/2783) in the ramucirumab group (only 1 case in each control group). Fortunately, in the reported cases of RCTs, these events were all within the safe range that could be controlled by medical treatment, with no unexpected safety events reported.

VEGF has been proved to be essential for the occurrence, development, and metastasis of cancer, and its mediated carcinogenesis is mainly completed by affecting the formation of new blood vessels and vascular permeability. In the past few decades, many anticancer drugs that target VEGF receptors or subtypes or signaling pathways have been approved by the United States Food and Drug Administration. These targeted drugs can be divided into the following categories according to the mechanism of action: (1) block the binding of ligands to receptors, such as bevacizumab, which targets VEGF-A, (2) block signaling through VEGFR, such as ramucirumab, which targets VEGFR2, and (3) tyrosine kinase inhibitors that block VEGFR1, VEGFR2, and VEGFR3 kinase activity, such as pazopanib and lenvatinib [41, 42]. The first approved antiangiogenic drug, bevacizumab, was used in clinical practice as early as 15 years ago. Although bevacizumab in combination with chemotherapy was well tolerated in multiple cancers and resulted in more beneficial PFS, no OS benefit was reported. Therefore, a large number of clinical trials of ramucirumab have been carried out, and its efficacy and safety have been reported in 11 phase 3 global RCTs. Two previous meta-analyses of 6 global trials have been published [43, 44]. However, these two studies did not report the results of PFS, OS, ORR, or DCR. The present study was a comprehensive update and meta-analysis of 11 global, double-blind, phase 3 RCTs, which were treated with standard regimens plus ramucirumab or placebo for advanced or metastatic cancer. This study represented one of the largest meta-analysis of antiangiogenic therapy for advanced cancer. A total of 4078 patients treated with ramucirumab and 3332 patients treated with placebo were included in this evaluation, which was a great strength and provided high-quality evidence for the safety and efficacy of ramucirumab therapy. A potential limitation of this meta-analysis is that 3 of these RCTs only reported once on different types of cancer. Encouragingly, the global RCTs on ramucirumab are still ongoing. Although the current RCTs have confirmed the important role of the VEGFR-2 signaling pathway as a therapeutic target in advanced cancer; furthermore, studies also need to explore the potential predictive biomarkers of ramucirumab and provide guidance for clinical treatment.

## 5. Conclusion

In conclusion, the meta-analysis showed that standard regimens combined with ramucirumab resulted in more beneficial PFS and OS outcomes in advanced NSCLC, HCC, gastric cancer, or GEJ adenocarcinoma. The importance of the results for clinical practice was that the incidence of hypertension, proteinuria, bleeding, liver injury/failure, VTE, and GI perforation were consistent with reported TEAEs of special interest associated with the angiogenesis inhibitor class. However, the authors do not observe an increased incidence associated with ramucirumab for high-grade bleeding, ATE, IRR, renal failure, fistula, or healing complication across these RCTs. Also, these adverse events were largely controllable after dose adjustment and supportive treatment.

## Figures and Tables

**Table 1:** Ramucirumab double-blind phase 3 RCTs.

Study	*Cancer* types	Treatment exposure	Sample size	Trial registry number	Collection time
RAINBOW-Asia [27]	Advanced gastric or GEJ adenocarcinoma	RAM 8 mg/kg (days 1 and 15) + paclitaxel VS. PBO + paclitaxel	294/146	NCT02898077	2017.03.02 ∼ 2020.06.30
RELAY [28]	Advanced NSCLC	RAM 10 mg/kg (IV; Q2W) + erlotinib VS. PBO + erlotinib	224/225	NCT02411448	2016.01.28 ∼ 2018.02.1
REACH-2 [29]	Advanced HCC	RAM 8 mg/kg (IV; Q2W) after sorafenib + BSC VS. PBO after sorafenib + BSC	197/95	NCT02435433	2015.07.26 ∼ 2017.08.30
RANGE [30]	Locally advanced or metastatic UC	RAM 10 mg/kg + docetaxel 60 or 75 mg/m^2^ (IV; day 1, 21-day cycle) VS. PBO + docetaxel	263/267	NCT02426125	2015.07.20 ∼ 2017.04.04
RAINFALL [31]	Metastatic gastric or junctional adenocarcinoma	RAM 8 mg/kg (IV; days 1 and 8, Q21D) + cisplatin + capecitabine VS. PBO + cisplatin + capecitabine	326/319	NCT02314117	2015.01.28 ∼ 2016.09.16
RAINBOW [32]	Advanced gastric or GEJ adenocarcinoma	RAM 8 mg/kg (IV; days 1 and 15) + paclitaxel VS. PBO + paclitaxel	330/335	NCT01170663	2010.12.02 ∼ 2012.09.23
RAISE [33]	Metastatic colorectal carcinoma	RAM 8 mg/kg (IV; day 1, Q2W) + FOLFIRI VS. PBO + FOLFIRI	536/536	NCT01183780	2010.12.14 ∼ 2013.08.23
REVEL [34]	Stage IV non-small cell lung cancer	RAM 10 mg/kg + docetaxel (IV; day 1, 21-day cycle) VS. PBO + docetaxel	628/625	NCT01168973	2010.12.03 ∼ 2013.01.24
REACH [35]	Advanced hepatocellular carcinoma	RAM 8 mg/kg (IV; Q2W) after sorafenib + BSC VS. PBO after sorafenib + BSC	283/282	NCT01140347	2010.11.04 ∼ 2013.04.18
REGARD [36]	Advanced gastric or GEJ adenocarcinoma	RAM 8 mg/kg (IV; Q2W) after chemotherapy + BSC VS. PBO after chemotherapy + BSC	238/117	NCT00917384	2009.10.06 ∼ 2012.01.26
ROSE/TRIO-012 [37]	Metastatic breast cancer	RAM 10 mg/kg + docetaxel (IV; Q3W) VS. PBO + docetaxel	759/385	NCT00703326	2008.08 ∼ 2011.12

RCTs = randomized controlled trials, GEJ = gastroesophageal junction, UC = urothelial carcinoma, RAM = ramucirumab, PBO = placebo, VS. = versus, FOLFIRI = leucovorin (folinic acid), fluorouracil, and irinotecan, BSC = best supportive care, IV = intravenous, Q2W = every 2 weeks, Q3W = every 3 weeks, and Q21D = every 21 days.

**Table 2:** Characteristics in the intent-to-treat population of phase 3 RCTs.

Total	*RELAY*		*RANGE*		*RAINFALL*		*RAINBOW-Asia*		*REACH-2*		*REACH*		*REGARD*		*REVEL*		*RAINBOW*		*ROSE/TRIO-012*		*RAISE*	
	RAM 224	Con 225	RAM 263	Con 267	RAM 326	Con 319	RAM 294	Con 146	RAM 197	Con 95	RAM 283	Con 282	RAM 238	Con 117	RAM 628	Con 625	RAM 330	Con 335	RAM 759	Con 385	RAM 536	Con 536
*Age (years)*																						
Median (range)	65 (57–71)	64 (56–70)	65 (59–72)	66 (59–72)	60 (51–68)	62(54–68)	57 (22–84)	58(18–79)	64 (58–73)	64 (56–71)	64 (28–87)	62 (25–85)	60 (52–67)	60 (51–71)	62 (21–85)	61 (25–86)	61 (25–83)	61 (24–84)	54 (24–82)	54 (29–81)	62 (21–83)	62 (33–87)
<65*∗*	102(46)	114(51)	124(47)	115(43)	203 (62)	202 (63)	225 (77)	112 (77)	102(52)	49(52)	150 (53)	162 (57)	156 (66)	71 (61)	391 (62)	407 (65)	204 (62)	212 (63)	629 (83)	325 (84)	324 (60)	321 (60)
≥65*∗*	122 (54)	111 (49)	139 (53)	152 (57)	123 (38)	117 (37)	69 (23)	34 (23)	95(48)	46(48)	133 (47)	120 (43)	82 (34)	46 (39)	237 (38)	218 (35)	126 (38)	123 (37)	130 (17)	60 (16)	212 (40)	215 (40)
*Gender*																						
Male*∗*	83 (37)	83 (37)	213 (81)	215 (81)	214 (66)	215 (67)	205 (70)	96 (66)	154 (78)	79 (83)	236 (83)	242 (86)	169 (71)	79 (68)	419 (67)	415 (66)	229 (69)	243 (73)	0 (0)	0 (0)	289 (54)	326 (61)
Female*∗*	141 (63)	142 (63)	50 (19)	52 (19)	112 (34)	104 (33)	89 (30)	50 (34)	43 (22)	16 (17)	47 (17)	40 (14)	69 (29)	38 (32)	209 (33)	210 (34)	101 (31)	92 (27)	759(100)	385(100)	247 (46)	210 (39)
*Race*																						
White*∗*	52 (23)	48 (21)	203 (77)	204 (76)	256 (79)	264 (83)	0 (0)	0 (0)	60 (30)	31 (33)	139 (49)	137 (49)	181 (76)	91 (78)	526 (84)	503 (80)	208 (63)	199 (59)	676 (89)	341 (89)	405 (76)	410 (76)
Asian*∗*	172 (77)	174 (78)	54 (21)	61 (23)	38 (12)	31 (10)	294 (100)	146 (100)	102 (52)	45 (47)	131 (46)	135 (48)	39 (16)	17 (15)	74 (12)	86 (14)	110 (33)	121 (36)	83(11)	44(11)	111 (21)	103 (19)
Black/Other*∗*	0 (0)	3 (1)	4(2)	2 (<1)	15 (4)	14 (4)	0 (0)	0 (0)	1 (1)	2(2)	13 (5)	10(3)	18 (2)	9 (7)	27 (4)	36(6)	12 (4)	15 (5)	NR	NR	18(3)	18 (4)
NA*∗*	0 (0)	0 (0)	2 (<1)	0 (0)	17 (5)	10 (3)	0 (0)	0 (0)	34 (17)	17 (18)	0 (0)	0 (0)	0 (0)	0 (0)	1 (<1)	0 (0)	0 (0)	0 (0)	NR	NR	2 (<1)	5 (1)
*ECOG PS*																						
0*∗*	116 (52)	119 (53)	121 (46)	125 (47)	141 (43)	143 (45)	65 (22)	31 (21)	113 (57)	55 (58)	159 (56)	153 (54)	67 (28)	31 (26)	207 (33)	199 (32)	117 (35)	144 (43)	439 (58)	240 (62)	263 (49)	259 (48)
≥1*∗*	108 (48)	106 (47)	139 (53)	142 (53)	185 (57)	176 (55)	229 (78)	115 (79)	84 (43)	40 (42)	124 (44)	129 (46)	171 (72)	86 (74)	420 (67)	425 (68)	213 (65)	191 (57)	320 (42)	145 (38)	269 (50)	275 (53)
NA*∗*	0 (0)	0 (0)	3 (1)	0 (0)	0 (0)	0 (0)	0 (0)	0 (0)	0 (0)	0 (0)	0 (0)	0 (0)	0 (0)	0 (0)	1 (<1)	1 (<1)	0 (0)	0 (0)	0 (0)	0 (0)	4 (1)	2 (<1)

RCTs = randomized controlled trials, ECOG PS = eastern cooperative oncology group performance status, RAM = ramucirumab group, Con = control group, and NR = not reported or missing, ^*∗*^  = *n* (%).

**Table 3:** Meta analysis of TEAEs of special interest.

TEAEs of special interest	Studies, *n*	*RAM, n*		*Con, n*		OR (95% CI)	*p* value
		Events	Total	Events	Total		
*Bleeding/haemorrhage*	10						
Grade 1-2		1304	3782	503	3028	2.72 [2.41, 3.07]	<0.00001
Grade ≧3		107	3782	87	3028	1.02 [0.76, 1.37]	0.89

*Hypertension*	10						
Grade 1-2		600	3782	190	3028	2.57 [2.16, 3.05]	<0.00001
Grade ≧3		379	3782	88	3028	3.86 [3.04, 4.89]	<0.00001

*Proteinuria*	10						
Grade 1-2		486	3782	153	3028	2.94 [2.42, 3.57]	<0.00001
Grade ≧3		55	3782	4	3028	6.46 [3.03, 13.78]	<0.00001

*CHF*	9						
Grade 1-2		25	3546	12	2913	1.59 [0.80, 3.15]	0.19
Grade ≧3		19	3546	9	2913	1.62 [0.78, 3.37]	0.20

*Liver injury/failure*	5						
Grade 1-2		361	1623	170	1373	1.73 [1.40, 2.15]	<0.00001
Grade ≧3		155	1623	125	1373	1.01 [0.78, 1.31]	0.92

*IRR*	10						
Grade 1-2		216	3717	118	2978	1.59 [0.86, 2.93]	0.06
Grade ≧3		31	3717	13	2978	1.59 [0.86, 2.93]	0.14

*VTE*	10						
Grade 1-2		111	3782	132	3028	0.68 [0.53, 0.89]	0.004
Grade ≧3		79	3782	90	3028	0.76 [0.56, 1.03]	0.07

*ATE*	10						
Grade 1-2		44	3782	37	3028	0.97 [0.62, 1.50]	0.88
Grade ≧3		30	3782	29	3028	0.85 [0.51, 1.42]	0.54

*GI perforation*	8						
Grade 1-2		11	2753	5	2370	1.61 [0.62, 4.17]	0.33
Grade ≧3		35	2753	9	2370	3.24 [1.60, 6.57]	0.001

*GI haemorrhage*	10						
Grade 1-2		174	3288	85	2911	1.88 [1.44, 2.46]	<0.00001
Grade ≧3		66	3288	49	2911	1.17 [0.80, 1.70]	0.42

*Renal failure*	5						
Grade 1-2		55	2018	54	2016	1.02 [0.69, 1.49]	0.94
Grade ≧3		23	2018	13	2016	1.75 [0.89, 3.43]	0.10

*Epistaxis*	4						
Grade 1-2		393	1574	149	1466	3.19 [2.59, 3.92]	<0.00001
Grade ≧3		3	1574	1	1466	1.77 [0.26, 12.18]	0.56

*Fistula*	5						
Grade 1-2		5	1270	1	895	1.51 [0.35, 6.50]	0.58
Grade ≧3		5	1270	1	895	1.87 [0.45, 7.87]	0.39

*Healing complication*	4						
Grade 1-2		5	1114	2	961	2.00 [0.50, 8.02]	0.33
Grade ≧3		1	1114	0	961	2.93 [0.12, 72.32]	0.51

*Pulmonary haemorrhage*	5						
Grade 1-2		75	1645	52	1529	1.43 [0.99, 2.05]	0.06
Grade ≧3		11	1645	11	1529	0.93 [0.42, 2.07]	0.87

TEAEs = treatment-emergent adverse events, RAM = ramucirumab, Con = control, OR = odds ratio, CI = confidence interval, CHF = congestive heart failure, IRR = infusion-related reaction, VTE = venous thromboembolic, ATE = arterial thromboembolic, and GI = gastrointestinal.

**Table 4:** Meta analysis of TEAEs.

TEAEs	Studies, *n*	*RAM, n*		*Con, n*		OR (95% CI)	*p* value
		Events	Total	Events	Total		
*Fatigue*	9						
Grade 1-2		1686	3526	1269	2923	1.14 [1.03, 1.27]	0.01
Grade ≧3		383	3526	224	2923	1.44 [1.21, 1.71]	<0.00001

*Hypertension*	5						
Grade 1-2		174	1422	63	1279	2.55 [1.89, 3.44]	<0.00001
Grade ≧3		131	1422	29	1279	4.25 [1.57, 11.53]	0.004

*Neuropathy*	4						
Grade 1-2		349	1741	320	1740	1.12 [0.94, 1.33]	0.21
Grade≧3		49	1741	30	1740	1.66 [1.05, 2.62]	0.03

*Decreased appetite*	11						
Grade 1-2		1098	4040	743	3293	1.34 [1.20, 1.50]	<0.00001
Grade≧3		143	4040	55	3293	2.10 [1.52, 2.90]	<0.00001

*Abdominal pain*	9						
Grade 1-2		546	3067	451	2686	1.06 [0.82, 1.37]	0.65
Grade ≧3		89	3067	69	2686	1.13 [0.82, 1.56]	0.45

*Nausea*	9						
Grade 1-2		966	3052	850	2796	1.13 [1.01, 1.27]	0.04
Grade ≧3		52	3052	61	2796	0.84 [0.57, 1.22]	0.36

*Alopecia*	6						
Grade 1-2		643	2255	626	2110	0.94 [0.71, 1.24]	0.66
Grade ≧3		0	3782	0	3028	0.34 [0.04, 3.26]	0.35

*Diarrhoea*	9						
Grade 1-2		887	3052	823	2796	1.11 [0.86, 1.44]	0.41
Grade ≧3		144	3052	107	2796	1.52 [0.95, 2.43]	0.08

*Epistaxis*	6						
Grade 1-2		596	2128	151	1622	3.58 [2.94, 4.36]	<0.00001
Grade ≧3		1	2128	3	1622	1.53 [0.06, 37.57]	0.80

*Vomiting*	9						
Grade 1-2		593	3067	495	2686	1.09 [0.95, 1.25]	0.21
Grade≧3		70	3067	80	2686	0.78 [0.56, 1.08]	0.13

*Peripheral oedema*	5						
Grade 1-2		433	1957	206	1846	2.28 [1.90, 2.73]	<0.00001
Grade ≧3		10	1957	3	1846	2.37 [0.74, 7.58]	0.15

*Constipation*	8						
Grade 1-2		481	2744	414	2371	1.01 [0.87, 1.17]	0.89
Grade ≧3		9	2744	20	2371	0.41 [0.19, 0.88]	0.02

*Stomatitis*	6						
Grade 1-2		871	2714	420	2347	2.00 [1.57, 2.54]	<0.00001
Grade≧3		108	2714	31	2347	2.92 [1.94, 4.40]	<0.00001

*Pyrexia*	8						
Grade 1-2		427	2729	265	2481	1.55 [1.32, 1.83]	<0.00001
Grade ≧3		11	2729	7	2481	1.41 [0.57, 3.48]	0.45

*Proteinuria*	5						
Grade 1-2		270	1376	90	1240	2.89 [1.98, 4.21]	<0.00001
Grade ≧3		24	1376	2	1240	6.26 [2.05, 19.10]	0.001

*Dyspnoea*	6						
Grade 1-2		251	2174	245	1950	0.96 [0.79, 1.16]	0.65
Grade ≧3		44	2174	67	1950	0.82 [0.32, 2.09]	0.68

*Rash*	4						
Grade 1-2		137	1335	131	1347	1.06 [0.82, 1.37]	0.65
Grade ≧3		3	1335	8	1347	0.45 [0.14, 1.46]	0.18

*Weight decreased*	3						
Grade 1-2		8	1149	5	1002	1.37 [0.49, 3.80]	0.55
Grade ≧3		164	1149	106	1002	1.31 [0.83, 2.05]	0.25

*Cough*	6						
Grade 1-2		356	2274	261	2121	1.37 [1.15, 1.63]	0.0004
Grade ≧3		5	2274	7	2121	0.76 [0.27, 2.11]	0.60

*Back pain*	4						
Grade 1-2		143	1505	104	357	1.24 [0.95, 1.62]	0.12
Grade ≧3		12	1505	7	1357	1.67 [0.67, 4.16]	0.27

*Hypoalbuminaemia*	3						
Grade 1-2		150	897	56	750	2.05 [1.47, 2.87]	<0.0001
Grade ≧3		8	897	4	750	1.70 [0.54, 5.30]	0.36

*Myalgia*	2						
Grade 1-2		4	954	5	947	0.81 [0.23, 2.82]	0.74
Grade ≧3		112	954	76	947	1.52 [1.12, 2.07]	0.007

*Ascites*	3						
Grade 1-2		109	801	48	700	2.20 [1.53, 3.16]	<0.0001
Grade≧3		33	801	26	700	1.14 [0.67, 1.94]	0.63

*Headache*	5						
Grade 1-2		252	1957	148	1846	1.98 [1.17, 3.35]	0.01
Grade ≧3		8	1957	8	1846	0.93 [0.39, 2.22]	0.87

*Neutropenia*	6						
Grade 1-2		724	2816	591	2437	1.16 [1.02, 1.33]	0.03
Grade ≧3		858	2816	572	2437	1.67 [1.20, 2.33]	0.002

*Anaemia*	6						
Grade 1-2		424	2300	469	2170	0.83 [0.71, 0.96]	0.01
Grade ≧3		161	2593	179	2315	0.71 [0.56, 0.89]	0.003

*Leucopenia*	3						
Grade 1-2		218	1277	153	1262	1.50 [1.20, 1.88]	0.0004
Grade ≧3		190	1277	116	1262	1.69 [0.97, 2.94]	0.06

*Thrombocytopenia*	6						
Grade 1-2		381	2341	175	2331	2.47 [2.04, 3.00]	<0.00001
Grade ≧3		78	2341	26	2331	3.02 [1.94, 4.72]	<0.00001

**Table 5:** Subgroup meta-analysis of TEAEs of special interest based on different treatment regimens.

	Hypertension (95%CI)	Proteinuria (95%CI)	CHF (95%CI)	Renal failure (95%CI)	GI haemorrhage (95%CI)	Bleeding or haemorrhage (95%CI)	VTE (95%CI)	Liver injury or failure (95%CI)	Fistula (95%CI)	ATE (95%CI)	IRR (95%CI)	Epistaxis (95%CI)	GI perforation (95%CI)	Healing complication (95%CI)	Pulmonary haemorrhage (95%CI)
*DOC* *+* *R vs. DOC* *+* *P*															
Grade 1–2	2.66 [2.02, 3.51] ^*∗∗∗*^	4.12 [2.08, 8.14] ^*∗∗∗*^	1.42 [0.57, 3.57]	0.92 [0.45, 1.90]	1.76 [0.82, 3.78]	2.71 [2.23, 3.30] ^*∗∗∗*^	0.47 [0.30, 0.74] ^*∗∗*^	—	—	0.77 [0.40, 1.51]	0.95 [0.70, 1.28]	3.28 [2.25, 4.79] ^*∗∗∗*^	2.98 [0.60, 14.80]	—	1.05 [0.69, 1.60]
Grade ≧3	3.22 [1.95, 5.32] ^*∗∗∗*^	3.31 [0.37, 29.22]	2.71 [0.58, 12.76]	1.80 [0.38, 8.45]	1.28 [0.32, 5.21]	0.84 [0.46, 1.53]	0.51 [0.29, 0.90] ^*∗*^	—	—	1.10 [0.46, 2.61]	1.14 [0.55, 2.38]	1.97 [0.18, 21.83]	2.48 [0.48, 12.81]	—	0.99 [0.37, 2.64]

*PTX* *+* *R vs. PTX* *+* *P*															
Grade 1–2	2.16 [1.37, 3.40] ^*∗∗∗*^	2.19 [1.54, 3.12] ^*∗∗∗*^	2.42 [0.65, 9.00]	1.49 [0.68, 3.26]	1.40 [0.83, 2.34]	2.93 [2.16, 3.96] ^*∗∗∗*^	0.72 [0.31, 1.71]	1.57 [1.13, 2.16] ^*∗∗*^	2.50 [0.12, 52.32]	1.30 [0.31, 5.52]	1.80 [0.84, 3.90]	—	0.69 [0.09, 5.40]	—	—
Grade ≧3	3.30 [1.96, 5.57] ^*∗∗∗*^	4.69 [0.86, 25.68]	1.38 [0.28, 6.81]	2.03 [0.50, 8.19]	1.70 [0.77, 3.75]	1.38 [0.69, 2.75]	0.70 [0.29, 1.68]	1.02 [0.60, 1.76]	1.49 [0.06, 36.86]	0.85 [0.21, 3.41]	3.56 [0.41, 30.54]	—	9.17 [0.49, 170.95]	—	—

*BSC* *+* *R vs. BSC* *+* *P*															
Grade 1–2	2.08 [1.31, 3.30] ^*∗∗∗*^	3.45 [2.08, 5.74] ^*∗∗∗*^	0.33 [0.01, 8.16]	0.93 [0.44, 1.96]	1.65 [0.77, 3.52]	2.30 [1.62, 3.25] ^*∗∗∗*^	0.92 [0.41, 2.07]	2.32 [1.61, 3.33] ^*∗∗∗*^	0.81 [0.11, 6.17]	1.52 [0.44, 5.23]	2.35 [0.43, 12.77]	4.66 [1.37, 15.82] ^*∗*^	0.97 [0.09, 10.86]	0.33 [0.01, 8.16]	3.01 [0.84,10.80]
Grade ≧3	3.30 [1.94, 5.61] ^*∗∗∗*^	5.72 [1.08, 30.38] ^*∗*^	0.40 [0.05, 3.23]	2.01 [0.50, 8.14]	0.79 [0.41, 1.52]	0.99 [0.58, 1.67]	#0.33 [0.12, 0.94] ^*∗*^	0.94 [0.67, 1.32]	0.49 [0.03, 7.83]	1.35 [0.33, 5.53]	7.05 [0.36, 137.15]	1.46 [0.06, 36.13]	0.64 [0.14, 2.90]	—	0.74 [0.12, 4.57]

TEAEs = treatment-emergent adverse events, CI = confidence interval, DOC = docetaxel, PTX = paclitaxel, BSC = best supportive care, RAM = ramucirumab, *P* = placebo, CHF = congestive heart failure, IRR = = infusion-related reaction, VTE = venous thromboembolic, ATE = arterial thromboembolic, and GI = gastrointestinal.^#^ Higher risk in control group. ^^*∗*^^*P*  <  0.05, ^^*∗∗*^^*P*  <  0.01, and ^*∗∗∗*^*P* < 0.001.

## Data Availability

The original contributions presented in the study are included in the article.

## Supplementary Materials

Supplementary Materials Table S1: PubMed Search Strategy of studies. Table S2: Geographic region in the Intent-to-Treat Population of Phase 3 RCTs. Table S3: Detailed data for TEAEs of special interest. Table S4: Detailed data for TEAEs. Figure S1. Risk of bias graph: judgements about each risk of bias item presented as percentages across all included studies. Figure S2. Risk of bias summary: judgements about each risk of bias item for each included study.

## References

[1] Lin L., Li Z., Yan L., Liu Y., Yang H., Li H. (2021). Global, regional, and national cancer incidence and death for 29 cancer groups in 2019 and trends analysis of the global cancer burden, 1990-2019. *Journal of Hematology & Oncology*.

[2] Siegel R. L., Miller K. D., Fuchs H. E., Jemal A. (2021). Cancer statistics, 2021. *CA: A Cancer Journal for Clinicians*.

[3] Meric-Bernstam F., Larkin J., Tabernero J., Bonini C. (2021). Enhancing anti-tumour efficacy with immunotherapy combinations. *The Lancet*.

[4] Jiang X., Wang J., Deng X. (2020). The role of microenvironment in tumor angiogenesis. *Journal of Experimental & Clinical Cancer Research*.

[5] Ribatti D. (2021). The fundamental contribution of judah folkman in the setting of angiogenesis assays. *Methods in Molecular Biology*.

[6] Cimpean A. M., Raica M. (2021). Historical overview of in vivo and in vitro angiogenesis assays. *Methods in Molecular Biology*.

[7] Ribatti D., Tamma R. (2019). Hematopoietic growth factors and tumor angiogenesis. *Cancer Letters*.

[8] Boye A. (2021). A cytokine in turmoil: transforming growth factor beta in cancer. *Biomedicine & Pharmacotherapy*.

[9] Balsano R., Kruize Z., Lunardi M. (2022). Transforming growth factor-beta signaling in cancer-induced cachexia: from molecular pathways to the clinics. *Cells*.

[10] Albadari N., Deng S., Li W. (2019). The transcriptional factors HIF-1 and HIF-2 and their novel inhibitors in cancer therapy. *Expert Opinion on Drug Discovery*.

[11] Kong D., Zhou H., Neelakantan D. (2021). VEGF-C mediates tumor growth and metastasis through promoting EMT-epithelial breast cancer cell crosstalk. *Oncogene*.

[12] Rah B., Banday M. A., Bhat G. R. (2021). Evaluation of biomarkers, genetic mutations, and epigenetic modifications in early diagnosis of pancreatic cancer. *World Journal of Gastroenterology*.

[13] Gershtein E. S., Korotkova E. A., Petrosyan A. P., Suleymanov E. A., Stilidi I. S., Kushlinskii N. E. (2021). Prognostic significance of VEGF signaling system components and matrix metalloproteinases in blood serum of gastric cancer patients. *Klinicheskaia Laboratornaia Diagnostika*.

[14] Mohamed S. Y., Mohammed H. L., Ibrahim H. M., Mohamed E. M., Salah M. (2019). Role of VEGF, CD105, and CD31 in the prognosis of colorectal cancer cases. *Journal of Gastrointestinal Cancer*.

[15] Cai C., Wang X., Fu Q., Chen A. (2022). The VEGF expression associated with prognosis in patients with intrahepatic cholangiocarcinoma: a systematic review and meta-analysis. *World Journal of Surgical Oncology*.

[16] Liu C., Zhou X., Zhang Z., Guo Y. (2020). Correlation of gene polymorphisms of vascular endothelial growth factor with grade and prognosis of lung cancer. *BMC Medical Genetics*.

[17] Albalawi I. A., Mir R., Abu Duhier F. M. (2020). Genetic effects of vascular endothelial growth factor A (VEGF-A) and its association with disease progression in breast cancer population of Saudi arabia. *Asian Pacific Journal of Cancer Prevention*.

[18] Patel K. A., Patel B. M., Thobias A. R. (2020). Overexpression of VEGF165 is associated with poor prognosis of cervical cancer. *Journal of Obstetrics and Gynaecology Research*.

[19] Maryam N., Ahmed S. S., Alam R., Hanif M. U., Saleem M., Gul R. (2021). Role of serum VEGF-A biomarker for early diagnosis of ovarian cancer instead of CA-125. *Journal of Pakistan Medical Association*.

[20] Zhang C., Wang L., Xiong C., Zhao R., Liang H., Luo X. (2021). The role of vascular endothelial growth factor as a prognostic and clinicopathological marker in osteosarcoma: a systematic review and meta-analysis. *Journal of Orthopaedic Surgery and Research*.

[21] Song Y., Zhuang G., Li J., Zhang M. (2021). BAIAP2L2 facilitates the malignancy of prostate cancer (PCa) via VEGF and apoptosis signaling pathways. *Genes & Genomics*.

[22] Wang N., Wang R., Tang J. (2022). Calbindin S100A16 promotes renal cell carcinoma progression and angiogenesis via the VEGF/VEGFR2 signaling pathway. *Contrast Media and Molecular Imaging*.

[23] Khan U., Shah M. A. (2019). Ramucirumab for the treatment of gastric or gastro-esophageal junction cancer. *Expert Opinion on Biological Therapy*.

[24] Ajani J. A., D’Amico T. A., Bentrem D. J. (2019). Esophageal and esophagogastric junction cancers, version 2.2019, NCCN clinical practice guidelines in Oncology. *Journal of the National Comprehensive Cancer Network*.

[25] Mehta R., Kommalapati A., Kim R. D. (2020). <p>The impact of ramucirumab treatment on survival and quality of life in patients with gastric cancer<</p>. *Cancer Management and Research*.

[26] Yu J., Zhang Y., Leung L. H., Liu L., Yang F., Yao X. (2016). Efficacy and safety of angiogenesis inhibitors in advanced gastric cancer: a systematic review and meta-analysis. *Journal of Hematology & Oncology*.

[27] Xu R. H., Zhang Y., Pan H. (2021). Efficacy and safety of weekly paclitaxel with or without ramucirumab as second-line therapy for the treatment of advanced gastric or gastroesophageal junction adenocarcinoma (RAINBOW-Asia): a randomised, multicentre, double-blind, phase 3 trial. *The Lancet Gastroenterology & Hepatology*.

[28] Nakagawa K., Garon E. B., Seto T. (2019). RELAY Study Investigators. Ramucirumab plus erlotinib in patients with untreated, EGFR-mutated, advanced non-small-cell lung cancer (RELAY): a randomised, double-blind, placebo-controlled, phase 3 trial. *The Lancet Oncology*.

[29] Zhu A. X., Kang Y. K., Yen C. J. (2019). Ramucirumab after sorafenib in patients with advanced hepatocellular carcinoma and increased *α*-fetoprotein concentrations (REACH-2): a randomised, double-blind, placebo-controlled, phase 3 trial. *The Lancet Oncology*.

[30] Petrylak D. P., de Wit R., Chi K. N. (2020). RANGE study investigators. Ramucirumab plus docetaxel versus placebo plus docetaxel in patients with locally advanced or metastatic urothelial carcinoma after platinum-based therapy (RANGE): overall survival and updated results of a randomised, double-blind, phase 3 trial. *The Lancet Oncology*.

[31] Fuchs C. S., Shitara K., Di Bartolomeo M. (2019). RAINFALL Study Group. Ramucirumab with cisplatin and fluoropyrimidine as first-line therapy in patients with metastatic gastric or junctional adenocarcinoma (RAINFALL): a double-blind, randomised, placebo-controlled, phase 3 trial. *The Lancet Oncology*.

[32] Wilke H., Muro K., Van Cutsem E. (2014). Ramucirumab plus paclitaxel versus placebo plus paclitaxel in patients with previously treated advanced gastric or gastro-oesophageal junction adenocarcinoma (RAINBOW): a double-blind, randomised phase 3 trial. *The Lancet Oncology*.

[33] Tabernero J., Yoshino T., Cohn A. L. (2015). Ramucirumab versus placebo in combination with second-line FOLFIRI in patients with metastatic colorectal carcinoma that progressed during or after first-line therapy with bevacizumab, oxaliplatin, and a fluoropyrimidine (RAISE): a randomised, double-blind, multicentre, phase 3 study. *The Lancet Oncology*.

[34] Garon E. B., Ciuleanu T. E., Arrieta O. (2014). Ramucirumab plus docetaxel versus placebo plus docetaxel for second-line treatment of stage IV non-small-cell lung cancer after disease progression on platinum-based therapy (REVEL): a multicentre, double-blind, randomised phase 3 trial. *The Lancet*.

[35] Zhu A. X., Park J. O., Ryoo B. Y. (2015). Ramucirumab versus placebo as second-line treatment in patients with advanced hepatocellular carcinoma following first-line therapy with sorafenib (REACH): a randomised, double-blind, multicentre, phase 3 trial. *The Lancet Oncology*.

[36] Fuchs C. S., Tomasek J., Yong C. J. (2014). Ramucirumab monotherapy for previously treated advanced gastric or gastro-oesophageal junction adenocarcinoma (REGARD): an international, randomised, multicentre, placebo-controlled, phase 3 trial. *The Lancet*.

[37] Mackey J. R., Ramos-Vazquez M., Lipatov O. (2015). Primary results of ROSE/TRIO-12, a randomized placebo-controlled phase III trial evaluating the addition of ramucirumab to first-line docetaxel chemotherapy in metastatic breast cancer. *Journal of Clinical Oncology*.

[38] Ferrara R., Imbimbo M., Malouf R. (2020). Single or combined immune checkpoint inhibitors compared to first-lineplatinum-based chemotherapy with or without bevacizumab for people with advanced non-small cell lung cancer. *Cochrane Database of Systematic Reviews*.

[39] Conforti F., Pala L., Bagnardi V. (2020). EGFR-TKI plus anti-angiogenic drugs in EGFR-mutatednon-small cell lung cancer: a meta-analysis of randomized clinical trials. *JNCI Cancer Spectrum*.

[40] Chen Z., Jiang S., Li X. (2021). Efficacy and safety of anti-angiogenic drugs combined with erlotinib in the treatment of advanced non-small cell lung cancer: a meta-analysis of randomized clinical trials. *Annals of Palliative Medicine*.

[41] Zirlik K., Duyster J. (2018). Anti-angiogenics: current situation and future perspectives. *Oncol Res Treat*.

[42] Cheng K., Liu C. F., Rao G. W. (2021). Anti-angiogenic agents: a review on vascular endothelial growth factor receptor-2 (VEGFR-2) inhibitors. *Current Medicinal Chemistry*.

[43] Arnold D., Fuchs C. S., Tabernero J. (2017). Meta-analysis of individual patient safety data from six randomized, placebo-controlled trials with the antiangiogenic VEGFR2-binding monoclonal antibody ramucirumab. *Annals of Oncology*.

[44] Yen C. J., Muro K., Kim T. W. (2018). Ramucirumab safety in east asian patients: a meta-analysis of six global, randomized, double-blind, placebo-controlled, phase III clinical trials. *Journal of Global Oncology*.

