# Supplementary material for: Safety and Efficacy of Treatment with/without Ramucirumab in Advanced or Metastatic Cancer: A Meta-Analysis of 11 Global, Double-Blind, Phase 3 Randomized Controlled Trials
Source: J Oncol. 2022 Nov 21;2022:2476469. doi: 10.1155/2022/2476469 (PMC9705087; doi:10.1155/2022/2476469)
Supplement: Supplementary Materials — Table S1: PubMed Search Strategy of studies. Table S2: Geographic region in the Intent-to-Treat Population of Phase 3 RCTs. Table S3: Detailed data for TEAEs of special interest. Table S4: Detailed data for TEAEs. Figure S1. Risk of bias graph: judgements about each risk of bias item presented as percentages across all included studies. Figure S2. Risk of bias summary: judgements about each risk of bias item for each included study. [file 2476469.f1.zip › Figure S2.pdf]

	Random sequence generation (selection bias)	Allocation concealment (selection bias)	Blinding of participants and personnel (performance bias)	Blinding of outcome assessment (detection bias)	Incomplete outcome data (attrition bias)	Selective reporting (reporting bias)	Other bias
RAINBOW 2014	+	+	+	+	+	+	?
RAINBOW-Asia 2021	+	+	+	+	+	+	?
RAINFALL 2019	+	+	+	+	+	+	?
RAISE 2015	+	+	+	+	+	+	?
RANGE 2020	+	+	+	+	+	+	?
REACH 2015	+	+	+	+	+	+	?
REACH-2 2019	+	+	+	+	+	+	?
REGARD 2014	+	+	+	+	+	+	?
RELAY 2019	+	+	+	+	+	+	?
REVEL 2014	+	+	+	+	+	+	?
ROSE/TRIO-012 2015	+	+	+	+	+	+	?
