# Supplementary material for: Safety and Efficacy of Treatment with/without Ramucirumab in Advanced or Metastatic Cancer: A Meta-Analysis of 11 Global, Double-Blind, Phase 3 Randomized Controlled Trials
Source: J Oncol. 2022 Nov 21;2022:2476469. doi: 10.1155/2022/2476469 (PMC9705087; doi:10.1155/2022/2476469)
Supplement: Supplementary Materials — Table S1: PubMed Search Strategy of studies. Table S2: Geographic region in the Intent-to-Treat Population of Phase 3 RCTs. Table S3: Detailed data for TEAEs of special interest. Table S4: Detailed data for TEAEs. Figure S1. Risk of bias graph: judgements about each risk of bias item presented as percentages across all included studies. Figure S2. Risk of bias summary: judgements about each risk of bias item for each included study. [file 2476469.f1.zip › Table S1.docx]

**Table S1**. PubMed Search Strategy

| Search No. | Query | Items Found, n |
| --- | --- | --- |
| 1 | Search “carcinoma” [MeSH] | 699671 |
| 2 | ((((((carcinoma [Title/Abstract])) OR (carcinomas [Title/Abstract])) OR (cancer [Title/Abstract])) OR (cancers [Title/Abstract])) OR (tumor [Title/Abstract])) OR (tumors [Title/Abstract])) | 3229234 |
| 3 | Search “ramucirumab” [Supplementary Concept] | 1084 |
| 4 | Search ((ramucirumab [Title/Abstract])) OR (Cyramza [Title/Abstract]) | 1045 |
| 5 | Search (no. 1) OR no. 2 | 3343147 |
| 6 | Search (no. 3) OR no. 4 | 1084 |
| 7 | Search (no. 5) and no. 6 | 1084 |

Abbreviations: MeSH = medical subject heading.
