# Supplementary material for: Safety and Efficacy of Treatment with/without Ramucirumab in Advanced or Metastatic Cancer: A Meta-Analysis of 11 Global, Double-Blind, Phase 3 Randomized Controlled Trials
Source: J Oncol. 2022 Nov 21;2022:2476469. doi: 10.1155/2022/2476469 (PMC9705087; doi:10.1155/2022/2476469)
Supplement: Supplementary Materials — Table S1: PubMed Search Strategy of studies. Table S2: Geographic region in the Intent-to-Treat Population of Phase 3 RCTs. Table S3: Detailed data for TEAEs of special interest. Table S4: Detailed data for TEAEs. Figure S1. Risk of bias graph: judgements about each risk of bias item presented as percentages across all included studies. Figure S2. Risk of bias summary: judgements about each risk of bias item for each included study. [file 2476469.f1.zip › Table S2.docx]

**Table S2.** Geographic region in the Intent-to-Treat Population of Phase 3 RCTs

| Study | Geographic region (N= ramucirumab / control group) |
| --- | --- |
| RELAY | East Asia (166/170) and other (58/55). |
| RANGE | North America (24/24).  Europe and rest of the world (186/186).  East Asia (53/57). |
| RAINFALL | North America (52/37).  Europe (194/205).  Japan (32/28) and other (48/49). |
| RAINBOW-Asia | China (257/135).  Malaysia (18/7).  Philippines (6/2).  Thailand (13/2). |
| REACH-2 | Americas, Europe, Australia, Israel (101/50).  Asia, excluding Japan (55/27).  Japan (41/18). |
| REACH | North and South America (32/33).  Europe (125/123).  East Asia (126 /126). |
| REGARD | North America, Europe, Australia, New Zealand (165/80).  Asia (18 /8).  South and Central America, India, South Africa, Middle East (55 /29 ). |
| REVEL | East Asia (43/46) and other (585/579). |
| RAINBOW | Europe, Israel, Australia, and the USA (198/200).  Argentina, Brazil, Chile, and Mexico (23/21).  Japan, South Korea, Hong Kong, Singapore, and Taiwan (109/114). |
| ROSE/TRIO-012 | North or South America (182/91).  Europe, Australia, or New Zealand (486/244).  Asia, Middle East, or Africa (91/50). |
| RAISE | Europe (235/235).  North America (143/143) and other (158/158). |
