# Supplementary material for: Safety and Efficacy of Treatment with/without Ramucirumab in Advanced or Metastatic Cancer: A Meta-Analysis of 11 Global, Double-Blind, Phase 3 Randomized Controlled Trials
Source: J Oncol. 2022 Nov 21;2022:2476469. doi: 10.1155/2022/2476469 (PMC9705087; doi:10.1155/2022/2476469)
Supplement: Supplementary Materials — Table S1: PubMed Search Strategy of studies. Table S2: Geographic region in the Intent-to-Treat Population of Phase 3 RCTs. Table S3: Detailed data for TEAEs of special interest. Table S4: Detailed data for TEAEs. Figure S1. Risk of bias graph: judgements about each risk of bias item presented as percentages across all included studies. Figure S2. Risk of bias summary: judgements about each risk of bias item for each included study. [file 2476469.f1.zip › Table S3.docx]

**Table S3.** Detailed data for TEAEs of special interest

| **Bleeding or haemorrhage (Grade1-2)** | | | | | **Bleeding or haemorrhage (Grade>=3)** | | | | |
| --- | --- | --- | --- | --- | --- | --- | --- | --- | --- |
|  | **RAM** | | **Control** | |  | **RAM** | | **Control** | |
|  | Events | Total | Events | Total |  | Events | Total | Events | Total |
| RAINBOW 2014 | 123 | 327 | 51 | 329 | RAINBOW 2014 | 14 | 327 | 8 | 329 |
| RAINBOW-Asia 2021 | 91 | 293 | 23 | 145 | RAINBOW-Asia 2021 | 9 | 293 | 5 | 145 |
| RAINFALL 2019 | 71 | 323 | 32 | 315 | RAINFALL 2019 | 11 | 323 | 13 | 315 |
| RAISE 2015 | 219 | 529 | 111 | 528 | RAISE 2015 | 13 | 529 | 9 | 528 |
| RANGE 2020 | / | / | / | / | RANGE 2020 | / | / | / | / |
| REACH 2015 | 73 | 277 | 34 | 276 | REACH 2015 | 17 | 277 | 21 | 276 |
| REACH-2 2019 | 38 | 197 | 9 | 95 | REACH-2 2019 | 10 | 197 | 3 | 95 |
| REGARD 2014 | 30 | 236 | 9 | 115 | REGARD 2014 | 8 | 236 | 3 | 115 |
| RELAY 2019 | 117 | 221 | 55 | 225 | RELAY 2019 | 3 | 221 | 4 | 225 |
| REVEL 2014 | 181 | 627 | 94 | 618 | REVEL 2014 | 15 | 627 | 14 | 618 |
| ROSE/TRIO-012 2015 | 361 | 752 | 85 | 382 | ROSE/TRIO-012 2015 | 7 | 752 | 7 | 382 |
|  |  |  |  |  |  |  |  |  |  |
| **Total** | 1304 | 3782 | 503 | 3028 | **Total** | 107 | 3782 | 87 | 3028 |
| **Fixed model (95% CI)** | 2.72 [2.41, 3.07] | | | | **Fixed model (95% CI)** | 1.02 [0.76, 1.37] | | | |
| **Heterogeneity** | Chi² = 7.56, df = 9 (P = 0.58); I² = 0% | | | | **Heterogeneity** | Chi² = 5.62, df = 9 (P = 0.78); I² = 0% | | | |
| **Test for overall effect** | Z = 16.35 (P < 0.00001) | | | | **Test for overall effect** | Z = 0.14 (P = 0.89) | | | |
|  |  |  |  |  |  |  |  |  |  |
|  |  |  |  |  |  |  |  |  |  |
| **Hypertension (Grade1-2)** | | | | | **Hypertension (Grade>=3)** | | | | |
|  | **RAM** | | **Control** | |  | **RAM** | | **Control** | |
|  | Events | Total | Events | Total |  | Events | Total | Events | Total |
| RAINBOW 2014 | 34 | 327 | 10 | 329 | RAINBOW 2014 | 48 | 327 | 9 | 329 |
| RAINBOW-Asia 2021 | 47 | 293 | 17 | 145 | RAINBOW-Asia 2021 | 24 | 293 | 9 | 145 |
| RAINFALL 2019 | 38 | 323 | 18 | 315 | RAINFALL 2019 | 32 | 323 | 5 | 315 |
| RAISE 2015 | 79 | 529 | 30 | 528 | RAISE 2015 | 59 | 529 | 15 | 528 |
| RANGE 2020 | / | / | / | / | RANGE 2020 | / | / | / | / |
| REACH 2015 | 21 | 277 | 10 | 276 | REACH 2015 | 35 | 277 | 10 | 276 |
| REACH-2 2019 | 24 | 197 | 7 | 95 | REACH-2 2019 | 25 | 197 | 5 | 95 |
| REGARD 2014 | 38 | 236 | 9 | 115 | REGARD 2014 | 18 | 236 | 3 | 115 |
| RELAY 2019 | 48 | 221 | 15 | 225 | RELAY 2019 | 52 | 221 | 12 | 225 |
| REVEL 2014 | 68 | 627 | 30 | 618 | REVEL 2014 | 35 | 627 | 13 | 618 |
| ROSE/TRIO-012 2015 | 203 | 752 | 44 | 382 | ROSE/TRIO-012 2015 | 51 | 752 | 7 | 382 |
|  |  |  |  |  |  |  |  |  |  |
| **Total** | 600 | 3782 | 190 | 3028 | **Total** | 379 | 3782 | 88 | 3028 |
| **Fixed model (95% CI)** | 2.57 [2.16, 3.05] | | | | **Fixed model (95% CI)** | 3.86 [3.04, 4.89] | | | |
| **Heterogeneity** | Chi² = 8.41, df = 9 (P = 0.49); I² = 0% | | | | **Heterogeneity** | Chi² = 12.60, df = 9 (P = 0.18); I² = 29% | | | |
| **Test for overall effect** | Z = 10.69 (P < 0.00001) | | | | **Test for overall effect** | Z = 11.09 (P < 0.00001) | | | |
|  |  |  |  |  |  |  |  |  |  |
|  |  |  |  |  |  |  |  |  |  |
| **Proteinuria (Grade1-2)** | | | | | **Proteinuria (Grade>=3)** | | | | |
|  | **RAM** | | **Control** | |  | **RAM** | | **Control** | |
|  | Events | Total | Events | Total |  | Events | Total | Events | Total |
| RAINBOW 2014 | 51 | 327 | 20 | 329 | RAINBOW 2014 | 4 | 327 | 0 | 329 |
| RAINBOW-Asia 2021 | 93 | 293 | 30 | 145 | RAINBOW-Asia 2021 | 6 | 293 | 1 | 145 |
| RAINFALL 2019 | 54 | 323 | 31 | 315 | RAINFALL 2019 | 8 | 323 | 2 | 315 |
| RAISE 2015 | 74 | 529 | 23 | 528 | RAISE 2015 | 16 | 529 | 1 | 528 |
| RANGE 2020 | / | / | / | / | RANGE 2020 | / | / | / | / |
| REACH 2015 | 42 | 277 | 13 | 276 | REACH 2015 | 6 | 277 | 0 | 276 |
| REACH-2 2019 | 36 | 197 | 4 | 95 | REACH-2 2019 | 4 | 197 | 0 | 95 |
| REGARD 2014 | 7 | 236 | 3 | 115 | REGARD 2014 | 1 | 236 | 0 | 115 |
| RELAY 2019 | 70 | 221 | 19 | 225 | RELAY 2019 | 6 | 221 | 0 | 225 |
| REVEL 2014 | 21 | 627 | 5 | 618 | REVEL 2014 | 1 | 627 | 0 | 618 |
| ROSE/TRIO-012 2015 | 38 | 752 | 5 | 382 | ROSE/TRIO-012 2015 | 3 | 752 | 0 | 382 |
|  |  |  |  |  |  |  |  |  |  |
| **Total** | 486 | 3782 | 153 | 3028 | **Total** | 55 | 3782 | 4 | 3028 |
| **Fixed model (95% CI)** | 2.94 [2.42, 3.57] | | | | **Fixed model (95% CI)** | 6.46 [3.03, 13.78] | | | |
| **Heterogeneity** | Chi² = 16.63, df = 9 (P = 0.05); I² = 46% | | | | **Heterogeneity** | Chi² = 3.50, df = 9 (P = 0.94); I² = 0% | | | |
| **Test for overall effect** | Z = 10.89 (P < 0.00001) | | | | **Test for overall effect** | Z = 4.83 (P < 0.00001) | | | |
|  |  |  |  |  |  |  |  |  |  |
|  |  |  |  |  |  |  |  |  |  |
| **Liver injury or failure (Grade1-2)** | | | | | **Liver injury or failure (Grade>=3)** | | | | |
|  | **RAM** | | **Control** | |  | **RAM** | | **Control** | |
|  | Events | Total | Events | Total |  | Events | Total | Events | Total |
| RAINBOW 2014 | 39 | 327 | 20 | 329 | RAINBOW 2014 | 15 | 327 | 13 | 329 |
| RAINBOW-Asia 2021 | 163 | 293 | 70 | 145 | RAINBOW-Asia 2021 | 20 | 293 | 11 | 145 |
| RAINFALL 2019 | / | / | / | / | RAINFALL 2019 | / | / | / | / |
| RAISE 2015 | 35 | 529 | 29 | 528 | RAISE 2015 | 26 | 529 | 21 | 528 |
| RANGE 2020 | / | / | / | / | RANGE 2020 | / | / | / | / |
| REACH 2015 | 82 | 277 | 38 | 276 | REACH 2015 | 58 | 277 | 65 | 276 |
| REACH-2 2019 | 42 | 197 | 13 | 95 | REACH-2 2019 | 36 | 197 | 15 | 95 |
| REGARD 2014 | / | / | / | / | REGARD 2014 | / | / | / | / |
| RELAY 2019 | / | / | / | / | RELAY 2019 | / | / | / | / |
| REVEL 2014 | / | / | / | / | REVEL 2014 | / | / | / | / |
| ROSE/TRIO-012 2015 | / | / | / | / | ROSE/TRIO-012 2015 | / | / | / | / |
|  |  |  |  |  |  |  |  |  |  |
| **Total** | 361 | 1623 | 170 | 1373 | **Total** | 155 | 1623 | 125 | 1373 |
| **Fixed model (95% CI)** | 1.73 [1.40, 2.15] | | | | **Fixed model (95% CI)** | 1.01 [0.78, 1.31] | | | |
| **Heterogeneity** | Chi² = 7.50, df = 4 (P = 0.11); I² = 47% | | | | **Heterogeneity** | Chi² = 1.60, df = 4 (P = 0.81); I² = 0% | | | |
| **Test for overall effect** | Z = 4.97 (P < 0.00001) | | | | **Test for overall effect** | Z = 0.10 (P = 0.92) | | | |
|  |  |  |  |  |  |  |  |  |  |
|  |  |  |  |  |  |  |  |  |  |
| **Infusion-related reaction (Grade1-2)** | | | | | **Infusion-related reaction (Grade>=3)** | | | | |
|  | **RAM** | | **Control** | |  | **RAM** | | **Control** | |
|  | Events | Total | Events | Total |  | Events | Total | Events | Total |
| RAINBOW 2014 | 17 | 327 | 12 | 329 | RAINBOW 2014 | 2 | 327 | 0 | 329 |
| RAINBOW-Asia 2021 | 14 | 293 | 2 | 145 | RAINBOW-Asia 2021 | 2 | 293 | 0 | 145 |
| RAINFALL 2019 | / | / | / | / | RAINFALL 2019 | / | / | / | / |
| RAISE 2015 | 27 | 529 | 14 | 528 | RAISE 2015 | 4 | 529 | 2 | 528 |
| RANGE 2020 | 8 | 258 | 7 | 265 | RANGE 2020 | 1 | 258 | 0 | 265 |
| REACH 2015 | 17 | 277 | 2 | 276 | REACH 2015 | 3 | 277 | 0 | 276 |
| REACH-2 2019 | 17 | 197 | 3 | 95 | REACH-2 2019 | 0 | 197 | 0 | 95 |
| REGARD 2014 | 1 | 236 | 2 | 115 | REGARD 2014 | 0 | 236 | 0 | 115 |
| RELAY 2019 | 6 | 221 | 4 | 225 | RELAY 2019 | 0 | 221 | 0 | 225 |
| REVEL 2014 | 23 | 627 | 28 | 618 | REVEL 2014 | 5 | 627 | 4 | 618 |
| ROSE/TRIO-012 2015 | 86 | 752 | 44 | 382 | ROSE/TRIO-012 2015 | 14 | 752 | 7 | 382 |
|  |  |  |  |  |  |  |  |  |  |
| **Total** | 216 | 3717 | 118 | 2978 | **Total** | 31 | 3717 | 13 | 2978 |
| **Random model (95% CI)** | 1.48 [0.99, 2.22] | | | | **Fixed model (95% CI)** | 1.59 [0.86, 2.93] | | | |
| **Heterogeneity** | Tau² = 0.18; Chi² = 18.43, df = 9 (P = 0.03); I² = 51% | | | | **Heterogeneity** | Chi² = 2.90, df = 6 (P = 0.82); I² = 0% | | | |
| **Test for overall effect** | Z = 1.90 (P = 0.06) | | | | **Test for overall effect** | Z = 1.47 (P = 0.14) | | | |
|  |  |  |  |  |  |  |  |  |  |
|  |  |  |  |  |  |  |  |  |  |
| **Gastrointestinal haemorrhage (Grade1-2)** | | | | | **Gastrointestinal haemorrhage (Grade>=3)** | | | | |
|  | **RAM** | | **Control** | |  | **RAM** | | **Control** | |
|  | Events | Total | Events | Total |  | Events | Total | Events | Total |
| RAINBOW 2014 | 21 | 327 | 15 | 329 | RAINBOW 2014 | 12 | 327 | 5 | 329 |
| RAINBOW-Asia 2021 | 24 | 293 | 9 | 145 | RAINBOW-Asia 2021 | 8 | 293 | 4 | 145 |
| RAINFALL 2019 | 15 | 323 | 6 | 315 | RAINFALL 2019 | 9 | 323 | 10 | 315 |
| RAISE 2015 | 55 | 529 | 30 | 528 | RAISE 2015 | 10 | 529 | 6 | 528 |
| RANGE 2020 | 1 | 258 | 0 | 265 | RANGE 2020 | 0 | 258 | 1 | 265 |
| REACH 2015 | 14 | 277 | 6 | 276 | REACH 2015 | 11 | 277 | 17 | 276 |
| REACH-2 2019 | 5 | 197 | 3 | 95 | REACH-2 2019 | 7 | 197 | 2 | 95 |
| REGARD 2014 | 2 | 236 | 1 | 115 | REGARD 2014 | 2 | 236 | 1 | 115 |
| RELAY 2019 | 20 | 221 | 5 | 225 | RELAY 2019 | 3 | 221 | 1 | 225 |
| REVEL 2014 | 17 | 627 | 10 | 618 | REVEL 2014 | 4 | 627 | 2 | 618 |
| ROSE/TRIO-012 2015 | / | / | / | / | ROSE/TRIO-012 2015 | / | / | / | / |
|  |  |  |  |  |  |  |  |  |  |
| **Total** | 174 | 3288 | 85 | 2911 | **Total** | 66 | 3288 | 49 | 2911 |
| **Fixed model (95% CI)** | 1.88 [1.44, 2.46] | | | | **Fixed model (95% CI)** | 1.17 [0.80, 1.70] | | | |
| **Heterogeneity** | Chi² = 6.41, df = 9 (P = 0.70); I² = 0% | | | | **Heterogeneity** | Chi² = 7.18, df = 9 (P = 0.62); I² = 0% | | | |
| **Test for overall effect** | Z = 4.66 (P < 0.00001) | | | | **Test for overall effect** | Z = 0.81 (P = 0.42) | | | |
|  |  |  |  |  |  |  |  |  |  |
|  |  |  |  |  |  |  |  |  |  |
| **Renal failure (Grade1-2)** | | | | | **Renal failure (Grade>=3)** | | | | |
|  | **RAM** | | **Control** | |  | **RAM** | | **Control** | |
|  | Events | Total | Events | Total |  | Events | Total | Events | Total |
| RAINBOW 2014 | 16 | 327 | 11 | 329 | RAINBOW 2014 | 6 | 327 | 3 | 329 |
| RAINBOW-Asia 2021 | / | / | / | / | RAINBOW-Asia 2021 | / | / | / | / |
| RAINFALL 2019 | / | / | / | / | RAINFALL 2019 | / | / | / | / |
| RAISE 2015 | 11 | 529 | 13 | 528 | RAISE 2015 | 7 | 529 | 5 | 528 |
| RANGE 2020 | 0 | 258 | 1 | 265 | RANGE 2020 | 1 | 258 | 0 | 265 |
| REACH 2015 | 14 | 277 | 15 | 276 | REACH 2015 | 6 | 277 | 3 | 276 |
| REACH-2 2019 | / | / | / | / | REACH-2 2019 | / | / | / | / |
| REGARD 2014 | / | / | / | / | REGARD 2014 | / | / | / | / |
| RELAY 2019 | / | / | / | / | RELAY 2019 | / | / | / | / |
| REVEL 2014 | 14 | 627 | 14 | 618 | REVEL 2014 | 3 | 627 | 2 | 618 |
| ROSE/TRIO-012 2015 | / | / | / | / | ROSE/TRIO-012 2015 | / | / | / | / |
|  |  |  |  |  |  |  |  |  |  |
| **Total** | 55 | 2018 | 54 | 2016 | **Total** | 23 | 2018 | 13 | 2016 |
| **Fixed model (95% CI)** | 1.02 [0.69, 1.49] | | | | **Fixed model (95% CI)** | 1.75 [0.89, 3.43] | | | |
| **Heterogeneity** | Chi² = 1.63, df = 4 (P = 0.80); I² = 0% | | | | **Heterogeneity** | Chi² = 0.38, df = 4 (P = 0.98); I² = 0% | | | |
| **Test for overall effect** | Z = 0.08 (P = 0.94) | | | | **Test for overall effect** | Z = 1.63 (P = 0.10) | | | |
|  |  |  |  |  |  |  |  |  |  |
|  |  |  |  |  |  |  |  |  |  |
| **Congestive heart failure (Grade1-2)** | | | | | **Congestive heart failure (Grade>=3)** | | | | |
|  | **RAM** | | **Control** | |  | **RAM** | | **Control** | |
|  | Events | Total | Events | Total |  | Events | Total | Events | Total |
| RAINBOW 2014 | 6 | 327 | 2 | 329 | RAINBOW 2014 | 2 | 327 | 2 | 329 |
| RAINBOW-Asia 2021 | 3 | 293 | 1 | 145 | RAINBOW-Asia 2021 | 2 | 293 | 0 | 145 |
| RAINFALL 2019 | 0 | 323 | 0 | 315 | RAINFALL 2019 | 1 | 323 | 0 | 315 |
| RAISE 2015 | 0 | 529 | 0 | 528 | RAISE 2015 | 4 | 529 | 3 | 528 |
| RANGE 2020 | / | / | / | / | RANGE 2020 | / | / | / | / |
| REACH 2015 | 0 | 277 | 1 | 276 | REACH 2015 | 0 | 277 | 1 | 276 |
| REACH-2 2019 | 0 | 197 | 0 | 95 | REACH-2 2019 | 1 | 197 | 1 | 95 |
| REGARD 2014 | / | / | / | / | REGARD 2014 | / | / | / | / |
| RELAY 2019 | 2 | 221 | 1 | 225 | RELAY 2019 | 2 | 221 | 0 | 225 |
| REVEL 2014 | 6 | 627 | 4 | 618 | REVEL 2014 | 5 | 627 | 1 | 618 |
| ROSE/TRIO-012 2015 | 8 | 752 | 3 | 382 | ROSE/TRIO-012 2015 | 2 | 752 | 1 | 382 |
|  |  |  |  |  |  |  |  |  |  |
| **Total** | 25 | 3546 | 12 | 2913 | **Total** | 19 | 3546 | 9 | 2913 |
| **Fixed model (95% CI)** | 1.59 [0.80, 3.15] | | | | **Fixed model (95% CI)** | 1.62 [0.78, 3.37] | | | |
| **Heterogeneity** | Chi² = 1.67, df = 5 (P = 0.89); I² = 0% | | | | **Heterogeneity** | Chi² = 3.91, df = 8 (P = 0.86); I² = 0% | | | |
| **Test for overall effect** | Z = 1.32 (P = 0.19) | | | | **Test for overall effect** | Z = 1.29 (P = 0.20) | | | |
|  |  |  |  |  |  |  |  |  |  |
|  |  |  |  |  |  |  |  |  |  |
| **Venous thromboembolic (Grade1-2)** | | | | | **Venous thromboembolic (Grade>=3)** | | | | |
|  | **RAM** | | **Control** | |  | **RAM** | | **Control** | |
|  | Events | Total | Events | Total |  | Events | Total | Events | Total |
| RAINBOW 2014 | 5 | 327 | 7 | 329 | RAINBOW 2014 | 8 | 327 | 11 | 329 |
| RAINBOW-Asia 2021 | 6 | 293 | 4 | 145 | RAINBOW-Asia 2021 | 1 | 293 | 1 | 145 |
| RAINFALL 2019 | 25 | 323 | 33 | 315 | RAINFALL 2019 | 19 | 323 | 22 | 315 |
| RAISE 2015 | 22 | 529 | 23 | 528 | RAISE 2015 | 22 | 529 | 11 | 528 |
| RANGE 2020 | / | / | / | / | RANGE 2020 | / | / | / | / |
| REACH 2015 | 4 | 277 | 0 | 276 | REACH 2015 | 2 | 277 | 4 | 276 |
| REACH-2 2019 | 2 | 197 | 1 | 95 | REACH-2 2019 | 0 | 197 | 1 | 95 |
| REGARD 2014 | 9 | 236 | 8 | 115 | REGARD 2014 | 3 | 236 | 5 | 115 |
| RELAY 2019 | 4 | 221 | 4 | 225 | RELAY 2019 | 3 | 221 | 5 | 225 |
| REVEL 2014 | 16 | 627 | 36 | 618 | REVEL 2014 | 11 | 627 | 18 | 618 |
| ROSE/TRIO-012 2015 | 18 | 752 | 16 | 382 | ROSE/TRIO-012 2015 | 10 | 752 | 12 | 382 |
|  |  |  |  |  |  |  |  |  |  |
| **Total** | 111 | 3782 | 132 | 3028 | **Total** | 79 | 3782 | 90 | 3028 |
| **Fixed model (95% CI)** | 0.68 [0.53, 0.89] | | | | **Fixed model (95% CI)** | 0.76 [0.56, 1.03] | | | |
| **Heterogeneity** | Chi² = 7.66, df = 9 (P = 0.57); I² = 0% | | | | **Heterogeneity** | Chi² = 12.50, df = 9 (P = 0.19); I² = 28% | | | |
| **Test for overall effect** | Z = 2.87 (P = 0.004) | | | | **Test for overall effect** | Z = 1.78 (P = 0.07) | | | |
|  |  |  |  |  |  |  |  |  |  |
|  |  |  |  |  |  |  |  |  |  |
| **Arterial thromboembolic (Grade1-2)** | | | | | **Arterial thromboembolic (Grade>=3)** | | | | |
|  | **RAM** | | **Control** | |  | **RAM** | | **Control** | |
|  | Events | Total | Events | Total |  | Events | Total | Events | Total |
| RAINBOW 2014 | 3 | 327 | 2 | 329 | RAINBOW 2014 | 3 | 327 | 3 | 329 |
| RAINBOW-Asia 2021 | 2 | 293 | 1 | 145 | RAINBOW-Asia 2021 | 1 | 293 | 1 | 145 |
| RAINFALL 2019 | 8 | 323 | 6 | 315 | RAINFALL 2019 | 4 | 323 | 8 | 315 |
| RAISE 2015 | 4 | 529 | 7 | 528 | RAISE 2015 | 4 | 529 | 6 | 528 |
| RANGE 2020 | / | / | / | / | RANGE 2020 | / | / | / | / |
| REACH 2015 | 2 | 277 | 3 | 276 | REACH 2015 | 0 | 277 | 1 | 276 |
| REACH-2 2019 | 2 | 197 | 0 | 95 | REACH-2 2019 | 3 | 197 | 1 | 95 |
| REGARD 2014 | 4 | 236 | 0 | 115 | REGARD 2014 | 3 | 236 | 0 | 115 |
| RELAY 2019 | 1 | 221 | 0 | 225 | RELAY 2019 | 1 | 221 | 0 | 225 |
| REVEL 2014 | 10 | 627 | 13 | 618 | REVEL 2014 | 6 | 627 | 8 | 618 |
| ROSE/TRIO-012 2015 | 8 | 752 | 5 | 382 | ROSE/TRIO-012 2015 | 5 | 752 | 1 | 382 |
|  |  |  |  |  |  |  |  |  |  |
| **Total** | 44 | 3782 | 37 | 3028 | **Total** | 30 | 3782 | 29 | 3028 |
| **Fixed model (95% CI)** | 0.97 [0.62, 1.50] | | | | **Fixed model (95% CI)** | 0.85 [0.51, 1.42] | | | |
| **Heterogeneity** | Chi² = 3.77, df = 9 (P = 0.93); I² = 0% | | | | **Heterogeneity** | Chi² = 4.28, df = 9 (P = 0.89); I² = 0% | | | |
| **Test for overall effect** | Z = 0.15 (P = 0.88) | | | | **Test for overall effect** | Z = 0.61 (P = 0.54) | | | |
|  |  |  |  |  |  |  |  |  |  |
|  |  |  |  |  |  |  |  |  |  |
| **Gastrointestinal perforation (Grade1-2)** | | | | | **Gastrointestinal perforation (Grade>=3)** | | | | |
|  | **RAM** | | **Control** | |  | **RAM** | | **Control** | |
|  | Events | Total | Events | Total |  | Events | Total | Events | Total |
| RAINBOW 2014 | 0 | 327 | 1 | 329 | RAINBOW 2014 | 4 | 327 | 0 | 329 |
| RAINBOW-Asia 2021 | 1 | 293 | 0 | 145 | RAINBOW-Asia 2021 | 0 | 293 | 0 | 145 |
| RAINFALL 2019 | 1 | 323 | 1 | 315 | RAINFALL 2019 | 13 | 323 | 1 | 315 |
| RAISE 2015 | 0 | 529 | 0 | 528 | RAISE 2015 | 9 | 529 | 3 | 528 |
| RANGE 2020 | / | / | / | / | RANGE 2020 | / | / | / | / |
| REACH 2015 | / | / | / | / | REACH 2015 | / | / | / | / |
| REACH-2 2019 | 0 | 197 | 0 | 95 | REACH-2 2019 | 2 | 197 | 2 | 95 |
| REGARD 2014 | 2 | 236 | 1 | 115 | REGARD 2014 | 2 | 236 | 1 | 115 |
| RELAY 2019 | 1 | 221 | 0 | 225 | RELAY 2019 | 0 | 221 | 0 | 225 |
| REVEL 2014 | 6 | 627 | 2 | 618 | REVEL 2014 | 5 | 627 | 2 | 618 |
| ROSE/TRIO-012 2015 | / | / | / | / | ROSE/TRIO-012 2015 | / | / | / | / |
|  |  |  |  |  |  |  |  |  |  |
| **Total** | 11 | 2753 | 5 | 2370 | **Total** | 35 | 2753 | 9 | 2370 |
| **Fixed model (95% CI)** | 1.61 [0.62, 4.17] | | | | **Fixed model (95% CI)** | 3.24 [1.60, 6.57] | | | |
| **Heterogeneity** | Chi² = 1.94, df = 5 (P = 0.86); I² = 0% | | | | **Heterogeneity** | Chi² = 6.98, df = 5 (P = 0.22); I² = 28% | | | |
| **Test for overall effect** | Z = 0.97 (P = 0.33) | | | | **Test for overall effect** | Z = 3.26 (P = 0.001) | | | |
|  |  |  |  |  |  |  |  |  |  |
|  |  |  |  |  |  |  |  |  |  |
| **Epistaxis (Grade1-2)** | | | | | **Epistaxis (Grade>=3)** | | | | |
|  | **RAM** | | **Control** | |  | **RAM** | | **Control** | |
|  | Events | Total | Events | Total |  | Events | Total | Events | Total |
| RAINBOW 2014 | / | / | / | / | RAINBOW 2014 | / | / | / | / |
| RAINBOW-Asia 2021 | / | / | / | / | RAINBOW-Asia 2021 | / | / | / | / |
| RAINFALL 2019 | / | / | / | / | RAINFALL 2019 | / | / | / | / |
| RAISE 2015 | 177 | 529 | 79 | 528 | RAISE 2015 | 0 | 529 | 0 | 528 |
| RANGE 2020 | / | / | / | / | RANGE 2020 | / | / | / | / |
| REACH 2015 | / | / | / | / | REACH 2015 | / | / | / | / |
| REACH-2 2019 | 26 | 197 | 3 | 95 | REACH-2 2019 | 1 | 197 | 0 | 95 |
| REGARD 2014 | / | / | / | / | REGARD 2014 | / | / | / | / |
| RELAY 2019 | 74 | 221 | 27 | 225 | RELAY 2019 | 0 | 221 | 0 | 225 |
| REVEL 2014 | 116 | 627 | 40 | 618 | REVEL 2014 | 2 | 627 | 1 | 618 |
| ROSE/TRIO-012 2015 | / | / | / | / | ROSE/TRIO-012 2015 | / | / | / | / |
|  |  |  |  |  |  |  |  |  |  |
| **Total** | 393 | 1574 | 149 | 1466 | **Total** | 3 | 1574 | 1 | 1466 |
| **Fixed model (95% CI)** | 3.19 [2.59, 3.92] | | | | **Fixed model (95% CI)** | 1.77 [0.26, 12.18] | | | |
| **Heterogeneity** | Chi² = 1.25, df = 3 (P = 0.74); I² = 0% | | | | **Heterogeneity** | Chi² = 0.02, df = 1 (P = 0.88); I² = 0% | | | |
| **Test for overall effect** | Z = 10.89 (P < 0.00001) | | | | **Test for overall effect** | Z = 0.58 (P = 0.56) | | | |
|  |  |  |  |  |  |  |  |  |  |
|  |  |  |  |  |  |  |  |  |  |
| **Fistula (Grade1-2)** | | | | | **Fistula (Grade>=3)** | | | | |
|  | **RAM** | | **Control** | |  | **RAM** | | **Control** | |
|  | Events | Total | Events | Total |  | Events | Total | Events | Total |
| RAINBOW 2014 | / | / | / | / | RAINBOW 2014 | / | / | / | / |
| RAINBOW-Asia 2021 | 2 | 293 | 0 | 145 | RAINBOW-Asia 2021 | 1 | 293 | 0 | 145 |
| RAINFALL 2019 | 0 | 323 | 0 | 315 | RAINFALL 2019 | 2 | 323 | 0 | 315 |
| RAISE 2015 | / | / | / | / | RAISE 2015 | / | / | / | / |
| RANGE 2020 | / | / | / | / | RANGE 2020 | / | / | / | / |
| REACH 2015 | / | / | / | / | REACH 2015 | / | / | / | / |
| REACH-2 2019 | 1 | 197 | 0 | 95 | REACH-2 2019 | 0 | 197 | 0 | 95 |
| REGARD 2014 | 1 | 236 | 1 | 115 | REGARD 2014 | 1 | 236 | 1 | 115 |
| RELAY 2019 | 1 | 221 | 0 | 225 | RELAY 2019 | 1 | 221 | 0 | 225 |
| REVEL 2014 | / | / | / | / | REVEL 2014 | / | / | / | / |
| ROSE/TRIO-012 2015 | / | / | / | / | ROSE/TRIO-012 2015 | / | / | / | / |
|  |  |  |  |  |  |  |  |  |  |
| **Total** | 5 | 1270 | 1 | 895 | **Total** | 5 | 1270 | 1 | 895 |
| **Fixed model (95% CI)** | 1.51 [0.35, 6.50] | | | | **Fixed model (95% CI)** | 1.87 [0.45, 7.87] | | | |
| **Heterogeneity** | Chi² = 0.93, df = 3 (P = 0.82); I² = 0% | | | | **Heterogeneity** | Chi² = 1.40, df = 3 (P = 0.70); I² = 0% | | | |
| **Test for overall effect** | Z = 0.56 (P = 0.58) | | | | **Test for overall effect** | Z = 0.86 (P = 0.39) | | | |
|  |  |  |  |  |  |  |  |  |  |
|  |  |  |  |  |  |  |  |  |  |
| **Healing complication (Grade1-2)** | | | | | **Healing complication (Grade>=3)** | | | | |
|  | **RAM** | | **Control** | |  | **RAM** | | **Control** | |
|  | Events | Total | Events | Total |  | Events | Total | Events | Total |
| RAINBOW 2014 | / | / | / | / | RAINBOW 2014 | / | / | / | / |
| RAINBOW-Asia 2021 | 0 | 293 | 0 | 145 | RAINBOW-Asia 2021 | 0 | 293 | 0 | 145 |
| RAINFALL 2019 | 3 | 323 | 0 | 315 | RAINFALL 2019 | 1 | 323 | 0 | 315 |
| RAISE 2015 | / | / | / | / | RAISE 2015 | / | / | / | / |
| RANGE 2020 | / | / | / | / | RANGE 2020 | / | / | / | / |
| REACH 2015 | 0 | 277 | 1 | 276 | REACH 2015 | 0 | 277 | 0 | 276 |
| REACH-2 2019 | / | / | / | / | REACH-2 2019 | / | / | / | / |
| REGARD 2014 | / | / | / | / | REGARD 2014 | / | / | / | / |
| RELAY 2019 | 2 | 221 | 1 | 225 | RELAY 2019 | 0 | 221 | 0 | 225 |
| REVEL 2014 | / | / | / | / | REVEL 2014 | / | / | / | / |
| ROSE/TRIO-012 2015 | / | / | / | / | ROSE/TRIO-012 2015 | / | / | / | / |
|  |  |  |  |  |  |  |  |  |  |
| **Total** | 5 | 1114 | 2 | 961 | **Total** | 1 | 1114 | 0 | 961 |
| **Fixed model (95% CI)** | 2.00 [0.50, 8.02] | | | | **Fixed model (95% CI)** | 2.93 [0.12, 72.32] | | | |
| **Heterogeneity** | Chi² = 1.88, df = 2 (P = 0.39); I² = 0% | | | | **Heterogeneity** | Not applicable | | | |
| **Test for overall effect** | Z = 0.98 (P = 0.33) | | | | **Test for overall effect** | Z = 0.66 (P = 0.51) | | | |
|  |  |  |  |  |  |  |  |  |  |
|  |  |  |  |  |  |  |  |  |  |
| **Pulmonary haemorrhage (Grade1-2)** | | | | | **Pulmonary haemorrhage (Grade>=3)** | | | | |
|  | **RAM** | | **Control** | |  | **RAM** | | **Control** | |
|  | Events | Total | Events | Total |  | Events | Total | Events | Total |
| RAINBOW 2014 | / | / | / | / | RAINBOW 2014 | / | / | / | / |
| RAINBOW-Asia 2021 | / | / | / | / | RAINBOW-Asia 2021 | / | / | / | / |
| RAINFALL 2019 | 1 | 323 | 0 | 315 | RAINFALL 2019 | 1 | 323 | 0 | 315 |
| RAISE 2015 | / | / | / | / | RAISE 2015 | / | / | / | / |
| RANGE 2020 | / | / | / | / | RANGE 2020 | / | / | / | / |
| REACH 2015 | 8 | 277 | 2 | 276 | REACH 2015 | 1 | 277 | 2 | 276 |
| REACH-2 2019 | 3 | 197 | 1 | 95 | REACH-2 2019 | 1 | 197 | 0 | 95 |
| REGARD 2014 | / | / | / | / | REGARD 2014 | / | / | / | / |
| RELAY 2019 | 14 | 221 | 3 | 225 | RELAY 2019 | 0 | 221 | 1 | 225 |
| REVEL 2014 | 49 | 627 | 46 | 618 | REVEL 2014 | 8 | 627 | 8 | 618 |
| ROSE/TRIO-012 2015 | / | / | / | / | ROSE/TRIO-012 2015 | / | / | / | / |
|  |  |  |  |  |  |  |  |  |  |
| **Total** | 75 | 1645 | 52 | 1529 | **Total** | 11 | 1645 | 11 | 1529 |
| **Fixed model (95% CI)** | 1.43 [0.99, 2.05] | | | | **Fixed model (95% CI)** | 0.93 [0.42, 2.07] | | | |
| **Heterogeneity** | Chi² = 7.75, df = 4 (P = 0.10); I² = 48% | | | | **Heterogeneity** | Chi² = 1.23, df = 4 (P = 0.87); I² = 0% | | | |
| **Test for overall effect** | Z = 1.91 (P = 0.06) | | | | **Test for overall effect** | Z = 0.17 (P = 0.87) | | | |
