# Supplementary material for: Safety and Efficacy of Treatment with/without Ramucirumab in Advanced or Metastatic Cancer: A Meta-Analysis of 11 Global, Double-Blind, Phase 3 Randomized Controlled Trials
Source: J Oncol. 2022 Nov 21;2022:2476469. doi: 10.1155/2022/2476469 (PMC9705087; doi:10.1155/2022/2476469)
Supplement: Supplementary Materials — Table S1: PubMed Search Strategy of studies. Table S2: Geographic region in the Intent-to-Treat Population of Phase 3 RCTs. Table S3: Detailed data for TEAEs of special interest. Table S4: Detailed data for TEAEs. Figure S1. Risk of bias graph: judgements about each risk of bias item presented as percentages across all included studies. Figure S2. Risk of bias summary: judgements about each risk of bias item for each included study. [file 2476469.f1.zip › Table S4.docx]

**Table S4.** Detailed data for TEAEs

| **Hypertension (Grade1-2)** | | | | | **Hypertension (Grade>=3)** | | | | |
| --- | --- | --- | --- | --- | --- | --- | --- | --- | --- |
|  | **RAM** | | **Control** | |  | **RAM** | | **Control** | |
|  | Events | Total | Events | Total |  | Events | Total | Events | Total |
| RAINBOW 2014 | 32 | 327 | 8 | 329 | RAINBOW 2014 | 46 | 327 | 8 | 329 |
| RAINBOW-Asia 2021 | 44 | 293 | 16 | 145 | RAINBOW-Asia 2021 | 21 | 293 | 9 | 145 |
| RAINFALL 2019 | 38 | 323 | 18 | 315 | RAINFALL 2019 | 32 | 323 | 5 | 315 |
| RAISE 2015 | / | / | / | / | RAISE 2015 | / | / | / | / |
| RANGE 2020 | 12 | 258 | 6 | 265 | RANGE 2020 | 12 | 258 | 0 | 265 |
| REACH 2015 | 0 | 0 | 0 | 0 | REACH 2015 | 0 | 0 | 0 | 0 |
| REACH-2 2019 | 0 | 0 | 0 | 0 | REACH-2 2019 | 0 | 0 | 0 | 0 |
| REGARD 2014 | 0 | 0 | 0 | 0 | REGARD 2014 | 0 | 0 | 0 | 0 |
| RELAY 2019 | 48 | 221 | 15 | 225 | RELAY 2019 | 52 | 221 | 12 | 225 |
| REVEL 2014 | / | / | / | / | REVEL 2014 | / | / | / | / |
| ROSE/TRIO-012 2015 | / | / | / | / | ROSE/TRIO-012 2015 | / | / | / | / |
| **Total** | 174 | 1422 | 63 | 1279 | **Total** | 131 | 1422 | 29 | 1279 |
| **Fixed model (95% CI)** | 2.55 [1.89, 3.44] | | | | **Random model (95% CI)** | 4.25 [1.57, 11.53] | | | |
| **Heterogeneity** | Chi² = 7.44, df = 4 (P = 0.11); I² = 46% | | | | **Heterogeneity** | Tau² = 0.71; Chi² = 13.37, df = 3 (P = 0.004); I² = 78% | | | |
| **Test for overall effect** | Z = 6.10 (P < 0.00001) | | | | **Test for overall effect** | Z = 2.84 (P = 0.004) | | | |
|  |  |  |  |  |  |  |  |  |  |
|  |  |  |  |  |  |  |  |  |  |
| **Fatigue (Grade1-2)** | | | | | **Fatigue (Grade>=3)** | | | | |
|  | **RAM** | | **Control** | |  | **RAM** | | **Control** | |
|  | Events | Total | Events | Total |  | Events | Total | Events | Total |
| RAINBOW 2014 | 147 | 327 | 126 | 329 | RAINBOW 2014 | 39 | 327 | 18 | 329 |
| RAINBOW-Asia 2021 | / | / | / | / | RAINBOW-Asia 2021 | / | / | / | / |
| RAINFALL 2019 | 165 | 323 | 159 | 315 | RAINFALL 2019 | 27 | 323 | 25 | 315 |
| RAISE 2015 | 244 | 529 | 234 | 528 | RAISE 2015 | 61 | 529 | 41 | 528 |
| RANGE 2020 | 84 | 258 | 80 | 265 | RANGE 2020 | 17 | 258 | 16 | 265 |
| REACH 2015 | 58 | 277 | 50 | 276 | REACH 2015 | 6 | 277 | 8 | 276 |
| REACH-2 2019 | 47 | 197 | 13 | 95 | REACH-2 2019 | 7 | 197 | 3 | 95 |
| REGARD 2014 | 84 | 236 | 46 | 115 | REGARD 2014 | 15 | 236 | 11 | 115 |
| RELAY 2019 | / | / | / | / | RELAY 2019 | / | / | / | / |
| REVEL 2014 | 343 | 627 | 309 | 618 | REVEL 2014 | 88 | 627 | 65 | 618 |
| ROSE/TRIO-012 2015 | 514 | 752 | 252 | 382 | ROSE/TRIO-012 2015 | 123 | 752 | 37 | 382 |
|  |  |  |  |  |  |  |  |  |  |
| **Total** | 1686 | 3526 | 1269 | 2923 | **Total** | 383 | 3526 | 224 | 2923 |
| **Fixed model (95% CI)** | 1.14 [1.03, 1.27] | | | | **Fixed model (95% CI)** | 1.44 [1.21, 1.71] | | | |
| **Heterogeneity** | Chi² = 6.28, df = 8 (P = 0.62); I² = 0% | | | | **Heterogeneity** | Chi² = 11.34, df = 8 (P = 0.18); I² = 29% | | | |
| **Test for overall effect** | Z = 2.51 (P = 0.01) | | | | **Test for overall effect** | Z = 4.05 (P < 0.0001) | | | |
|  |  |  |  |  |  |  |  |  |  |
|  |  |  |  |  |  |  |  |  |  |
| **Neuropathy (Grade1-2)** | | | | | **Neuropathy (Grade>=3)** | | | | |
|  | **RAM** | | **Control** | |  | **RAM** | | **Control** | |
|  | Events | Total | Events | Total |  | Events | Total | Events | Total |
| RAINBOW 2014 | 123 | 327 | 104 | 329 | RAINBOW 2014 | 27 | 327 | 15 | 329 |
| RAINBOW-Asia 2021 | / | / | / | / | RAINBOW-Asia 2021 | / | / | / | / |
| RAINFALL 2019 | / | / | / | / | RAINFALL 2019 | / | / | / | / |
| RAISE 2015 | 54 | 529 | 59 | 528 | RAISE 2015 | 5 | 529 | 2 | 528 |
| RANGE 2020 | 27 | 258 | 31 | 265 | RANGE 2020 | 0 | 258 | 3 | 265 |
| REACH 2015 | / | / | / | / | REACH 2015 | / | / | / | / |
| REACH-2 2019 | / | / | / | / | REACH-2 2019 | / | / | / | / |
| REGARD 2014 | / | / | / | / | REGARD 2014 | / | / | / | / |
| RELAY 2019 | / | / | / | / | RELAY 2019 | / | / | / | / |
| REVEL 2014 | 145 | 627 | 126 | 618 | REVEL 2014 | 17 | 627 | 10 | 618 |
| ROSE/TRIO-012 2015 | / | / | / | / | ROSE/TRIO-012 2015 | / | / | / | / |
|  |  |  |  |  |  |  |  |  |  |
| **Total** | 349 | 1741 | 320 | 1740 | **Total** | 49 | 1741 | 30 | 1740 |
| **Fixed model (95% CI)** | 1.12 [0.94, 1.33] | | | | **Fixed model (95% CI)** | 1.66 [1.05, 2.62] | | | |
| **Heterogeneity** | Chi² = 2.87, df = 3 (P = 0.41); I² = 0% | | | | **Heterogeneity** | Chi² = 2.99, df = 3 (P = 0.39); I² = 0% | | | |
| **Test for overall effect** | Z = 1.25 (P = 0.21) | | | | **Test for overall effect** | Z = 2.15 (P = 0.03) | | | |
|  |  |  |  |  |  |  |  |  |  |
|  |  |  |  |  |  |  |  |  |  |
| **Decreased appetite (Grade1-2)** | | | | | **Decreased appetite (Grade>=3)** | | | | |
|  | **RAM** | | **Control** | |  | **RAM** | | **Control** | |
|  | Events | Total | Events | Total |  | Events | Total | Events | Total |
| RAINBOW 2014 | 121 | 327 | 92 | 329 | RAINBOW 2014 | 10 | 327 | 13 | 329 |
| RAINBOW-Asia 2021 | 75 | 293 | 31 | 145 | RAINBOW-Asia 2021 | 6 | 293 | 2 | 145 |
| RAINFALL 2019 | 112 | 323 | 91 | 315 | RAINFALL 2019 | 21 | 323 | 10 | 315 |
| RAISE 2015 | 185 | 529 | 134 | 528 | RAISE 2015 | 13 | 529 | 10 | 528 |
| RANGE 2020 | 53 | 258 | 44 | 265 | RANGE 2020 | 4 | 258 | 1 | 265 |
| REACH 2015 | 56 | 277 | 48 | 276 | REACH 2015 | 5 | 277 | 2 | 276 |
| REACH-2 2019 | 43 | 197 | 18 | 95 | REACH-2 2019 | 3 | 197 | 1 | 95 |
| REGARD 2014 | 57 | 236 | 26 | 115 | REGARD 2014 | 8 | 236 | 4 | 115 |
| RELAY 2019 | 51 | 221 | 43 | 225 | RELAY 2019 | 6 | 221 | 4 | 225 |
| REVEL 2014 | 182 | 627 | 154 | 618 | REVEL 2014 | 14 | 627 | 8 | 618 |
| ROSE/TRIO-012 2015 | 163 | 752 | 62 | 382 | ROSE/TRIO-012 2015 | 53 | 752 | 0 | 382 |
|  |  |  |  |  |  |  |  |  |  |
| **Total** | 1098 | 4040 | 743 | 3293 | **Total** | 143 | 4040 | 55 | 3293 |
| **Fixed model (95% CI)** | 1.34 [1.20, 1.50] | | | | **Fixed model (95% CI)** | 2.10 [1.52, 2.90] | | | |
| **Heterogeneity** | Chi² = 3.71, df = 10 (P = 0.96); I² = 0% | | | | **Heterogeneity** | Chi² = 14.87, df = 10 (P = 0.14); I² = 33% | | | |
| **Test for overall effect** | Z = 5.31 (P < 0.00001) | | | | **Test for overall effect** | Z = 4.52 (P < 0.00001) | | | |
|  |  |  |  |  |  |  |  |  |  |
|  |  |  |  |  |  |  |  |  |  |
| **Abdominal pain (Grade1-2)** | | | | | **Abdominal pain (Grade>=3)** | | | | |
|  | **RAM** | | **Control** | |  | **RAM** | | **Control** | |
|  | Events | Total | Events | Total |  | Events | Total | Events | Total |
| RAINBOW 2014 | 98 | 327 | 87 | 329 | RAINBOW 2014 | 20 | 327 | 11 | 329 |
| RAINBOW-Asia 2021 | 35 | 293 | 23 | 145 | RAINBOW-Asia 2021 | 5 | 293 | 3 | 145 |
| RAINFALL 2019 | 67 | 323 | 60 | 315 | RAINFALL 2019 | 18 | 323 | 11 | 315 |
| RAISE 2015 | 122 | 529 | 120 | 528 | RAISE 2015 | 18 | 529 | 19 | 528 |
| RANGE 2020 | 10 | 258 | 8 | 265 | RANGE 2020 | 1 | 258 | 0 | 265 |
| REACH 2015 | 42 | 277 | 50 | 276 | REACH 2015 | 5 | 277 | 12 | 276 |
| REACH-2 2019 | 36 | 197 | 10 | 95 | REACH-2 2019 | 3 | 197 | 2 | 95 |
| REGARD 2014 | 68 | 236 | 32 | 115 | REGARD 2014 | 14 | 236 | 3 | 115 |
| RELAY 2019 | / | / | / | / | RELAY 2019 | / | / | / | / |
| REVEL 2014 | 68 | 627 | 61 | 618 | REVEL 2014 | 5 | 627 | 8 | 618 |
| ROSE/TRIO-012 2015 | / | / | / | / | ROSE/TRIO-012 2015 | / | / | / | / |
|  |  |  |  |  |  |  |  |  |  |
| **Total** | 546 | 3067 | 451 | 2686 | **Total** | 89 | 3067 | 69 | 2686 |
| **Fixed model (95% CI)** | 1.06 [0.82, 1.37] | | | | **Fixed model (95% CI)** | 1.13 [0.82, 1.56] | | | |
| **Heterogeneity** | Chi² = 4.02, df = 3 (P = 0.26); I² = 25% | | | | **Heterogeneity** | Chi² = 9.81, df = 8 (P = 0.28); I² = 18% | | | |
| **Test for overall effect** | Z = 0.45 (P = 0.65) | | | | **Test for overall effect** | Z = 0.76 (P = 0.45) | | | |
|  |  |  |  |  |  |  |  |  |  |
|  |  |  |  |  |  |  |  |  |  |
| **Nausea (Grade1-2)** | | | | | **Nausea (Grade>=3)** | | | | |
|  | **RAM** | | **Control** | |  | **RAM** | | **Control** | |
|  | Events | Total | Events | Total |  | Events | Total | Events | Total |
| RAINBOW 2014 | 109 | 327 | 100 | 329 | RAINBOW 2014 | 6 | 327 | 8 | 329 |
| RAINBOW-Asia 2021 | 53 | 293 | 22 | 145 | RAINBOW-Asia 2021 | 0 | 293 | 0 | 145 |
| RAINFALL 2019 | 187 | 323 | 161 | 315 | RAINFALL 2019 | 22 | 323 | 26 | 315 |
| RAISE 2015 | 249 | 529 | 257 | 528 | RAISE 2015 | 13 | 529 | 14 | 528 |
| RANGE 2020 | 55 | 258 | 35 | 265 | RANGE 2020 | 2 | 258 | 2 | 265 |
| REACH 2015 | 52 | 277 | 52 | 276 | REACH 2015 | 0 | 277 | 0 | 276 |
| REACH-2 2019 | 37 | 197 | 11 | 95 | REACH-2 2019 | 0 | 197 | 0 | 95 |
| REGARD 2014 | / | / | / | / | REGARD 2014 | / | / | / | / |
| RELAY 2019 | 55 | 221 | 42 | 225 | RELAY 2019 | 2 | 221 | 2 | 225 |
| REVEL 2014 | 169 | 627 | 170 | 618 | REVEL 2014 | 7 | 627 | 9 | 618 |
| ROSE/TRIO-012 2015 | / | / | / | / | ROSE/TRIO-012 2015 | / | / | / | / |
|  |  |  |  |  |  |  |  |  |  |
| **Total** | 966 | 3052 | 850 | 2796 | **Total** | 52 | 3052 | 61 | 2796 |
| **Fixed model (95% CI)** | 1.13 [1.01, 1.27] | | | | **Fixed model (95% CI)** | 0.84 [0.57, 1.22] | | | |
| **Heterogeneity** | Chi² = 11.31, df = 8 (P = 0.18); I² = 29% | | | | **Heterogeneity** | Chi² = 0.23, df = 5 (P = 1.00); I² = 0% | | | |
| **Test for overall effect** | Z = 2.05 (P = 0.04) | | | | **Test for overall effect** | Z = 0.92 (P = 0.36) | | | |
|  |  |  |  |  |  |  |  |  |  |
|  |  |  |  |  |  |  |  |  |  |
| **Alopecia (Grade1-2)** | | | | | **Alopecia (Grade>=3)** | | | | |
|  | **RAM** | | **Control** | |  | **RAM** | | **Control** | |
|  | Events | Total | Events | Total |  | Events | Total | Events | Total |
| RAINBOW 2014 | 107 | 327 | 126 | 329 | RAINBOW 2014 | 0 | 327 | 1 | 329 |
| RAINBOW-Asia 2021 | 83 | 293 | 55 | 145 | RAINBOW-Asia 2021 | 0 | 293 | 0 | 145 |
| RAINFALL 2019 | / | / | / | / | RAINFALL 2019 | / | / | / | / |
| RAISE 2015 | 155 | 529 | 165 | 528 | RAISE 2015 | 0 | 529 | 0 | 528 |
| RANGE 2020 | 61 | 258 | 80 | 265 | RANGE 2020 | 0 | 258 | 1 | 265 |
| REACH 2015 | / | / | / | / | REACH 2015 | / | / | / | / |
| REACH-2 2019 | / | / | / | / | REACH-2 2019 | / | / | / | / |
| REGARD 2014 | / | / | / | / | REGARD 2014 | / | / | / | / |
| RELAY 2019 | 75 | 221 | 44 | 225 | RELAY 2019 | 0 | 221 | 0 | 225 |
| REVEL 2014 | 162 | 627 | 156 | 618 | REVEL 2014 | 0 | 627 | 0 | 618 |
| ROSE/TRIO-012 2015 | / | / | / | / | ROSE/TRIO-012 2015 | / | / | / | / |
|  |  |  |  |  |  |  |  |  |  |
| **Total** | 643 | 2255 | 626 | 2110 | **Total** | 0 | 2255 | 2 | 2110 |
| **Fixed model (95% CI)** | 0.94 [0.71, 1.24] | | | | **Fixed model (95% CI)** | 0.34 [0.04, 3.26] | | | |
| **Heterogeneity** | Tau² = 0.09; Chi² = 20.26, df = 5 (P = 0.001); I² = 75% | | | | **Heterogeneity** | Chi² = 0.00, df = 1 (P = 0.99); I² = 0% | | | |
| **Test for overall effect** | Z = 0.44 (P = 0.66) | | | | **Test for overall effect** | Z = 0.94 (P = 0.35) | | | |
|  |  |  |  |  |  |  |  |  |  |
|  |  |  |  |  |  |  |  |  |  |
| **Diarrhoea (Grade1-2)** | | | | | **Diarrhoea (Grade>=3)** | | | | |
|  | **RAM** | | **Control** | |  | **RAM** | | **Control** | |
|  | Events | Total | Events | Total |  | Events | Total | Events | Total |
| RAINBOW 2014 | 94 | 327 | 71 | 329 | RAINBOW 2014 | 12 | 327 | 5 | 329 |
| RAINBOW-Asia 2021 | 65 | 293 | 20 | 145 | RAINBOW-Asia 2021 | 4 | 293 | 1 | 145 |
| RAINFALL 2019 | 98 | 323 | 93 | 315 | RAINFALL 2019 | 15 | 323 | 23 | 315 |
| RAISE 2015 | 159 | 529 | 220 | 528 | RAISE 2015 | 57 | 529 | 51 | 528 |
| RANGE 2020 | 53 | 258 | 41 | 265 | RANGE 2020 | 8 | 258 | 3 | 265 |
| REACH 2015 | 48 | 277 | 37 | 276 | REACH 2015 | 3 | 277 | 1 | 276 |
| REACH-2 2019 | 32 | 197 | 13 | 95 | REACH-2 2019 | 0 | 197 | 1 | 95 |
| REGARD 2014 | / | / | / | / | REGARD 2014 | / | / | / | / |
| RELAY 2019 | 139 | 221 | 157 | 225 | RELAY 2019 | 16 | 221 | 3 | 225 |
| REVEL 2014 | 199 | 627 | 171 | 618 | REVEL 2014 | 29 | 627 | 19 | 618 |
| ROSE/TRIO-012 2015 | / | / | / | / | ROSE/TRIO-012 2015 | / | / | / | / |
|  |  |  |  |  |  |  |  |  |  |
| **Total** | 887 | 3052 | 823 | 2796 | **Total** | 144 | 3052 | 107 | 2796 |
| **Random model (95% CI)** | 1.11 [0.86, 1.44] | | | | **Fixed model (95% CI)** | 1.52 [0.95, 2.43] | | | |
| **Heterogeneity** | Tau² = 0.11; Chi² = 32.94, df = 8 (P < 0.0001); I² = 76% | | | | **Heterogeneity** | Tau² = 0.21; Chi² = 16.03, df = 8 (P = 0.04); I² = 50% | | | |
| **Test for overall effect** | Z = 0.83 (P = 0.41) | | | | **Test for overall effect** | Z = 1.73 (P = 0.08) | | | |
|  |  |  |  |  |  |  |  |  |  |
|  |  |  |  |  |  |  |  |  |  |
| **Epistaxis (Grade1-2)** | | | | | **Epistaxis (Grade>=3)** | | | | |
|  | **RAM** | | **Control** | |  | **RAM** | | **Control** | |
|  | Events | Total | Events | Total |  | Events | Total | Events | Total |
| RAINBOW 2014 | 100 | 327 | 23 | 329 | RAINBOW 2014 | 0 | 327 | 0 | 329 |
| RAINBOW-Asia 2021 | 53 | 293 | 9 | 145 | RAINBOW-Asia 2021 | 0 | 293 | 0 | 145 |
| RAINFALL 2019 | / | / | / | / | RAINFALL 2019 | / | / | / | / |
| RAISE 2015 | / | / | / | / | RAISE 2015 | / | / | / | / |
| RANGE 2020 | 31 | 258 | 11 | 265 | RANGE 2020 | 0 | 258 | 0 | 265 |
| REACH 2015 | 38 | 277 | 17 | 276 | REACH 2015 | 0 | 277 | 0 | 276 |
| REACH-2 2019 | / | / | / | / | REACH-2 2019 | / | / | / | / |
| REGARD 2014 | / | / | / | / | REGARD 2014 | / | / | / | / |
| RELAY 2019 | 74 | 221 | 27 | 225 | RELAY 2019 | 0 | 221 | 0 | 225 |
| REVEL 2014 | / | / | / | / | REVEL 2014 | / | / | / | / |
| ROSE/TRIO-012 2015 | 300 | 752 | 64 | 382 | ROSE/TRIO-012 2015 | 1 | 752 | 0 | 382 |
|  |  |  |  |  |  |  |  |  |  |
| **Total** | 596 | 2128 | 151 | 1622 | **Total** | 1 | 2128 | 3 | 1622 |
| **Fixed model (95% CI)** | 3.58 [2.94, 4.36] | | | | **Fixed model (95% CI)** | 1.53 [0.06, 37.57] | | | |
| **Heterogeneity** | Chi² = 6.06, df = 5 (P = 0.30); I² = 18% | | | | **Heterogeneity** | Not applicable | | | |
| **Test for overall effect** | Z = 12.73 (P < 0.00001) | | | | **Test for overall effect** | Z = 0.26 (P = 0.80) | | | |
|  |  |  |  |  |  |  |  |  |  |
|  |  |  |  |  |  |  |  |  |  |
| **Vomiting (Grade1-2)** | | | | | **Vomiting (Grade>=3)** | | | | |
|  | **RAM** | | **Control** | |  | **RAM** | | **Control** | |
|  | Events | Total | Events | Total |  | Events | Total | Events | Total |
| RAINBOW 2014 | 78 | 327 | 56 | 329 | RAINBOW 2014 | 10 | 327 | 12 | 329 |
| RAINBOW-Asia 2021 | 46 | 293 | 21 | 145 | RAINBOW-Asia 2021 | 6 | 293 | 4 | 145 |
| RAINFALL 2019 | 121 | 323 | 100 | 315 | RAINFALL 2019 | 21 | 323 | 31 | 315 |
| RAISE 2015 | 139 | 529 | 131 | 528 | RAISE 2015 | 15 | 529 | 13 | 528 |
| RANGE 2020 | 27 | 258 | 25 | 265 | RANGE 2020 | 2 | 258 | 1 | 265 |
| REACH 2015 | 28 | 277 | 38 | 276 | REACH 2015 | 2 | 277 | 2 | 276 |
| REACH-2 2019 | 20 | 197 | 7 | 95 | REACH-2 2019 | 0 | 197 | 0 | 95 |
| REGARD 2014 | 47 | 236 | 29 | 115 | REGARD 2014 | 6 | 236 | 5 | 115 |
| RELAY 2019 | / | / | / | / | RELAY 2019 | / | / | / | / |
| REVEL 2014 | 87 | 627 | 88 | 618 | REVEL 2014 | 8 | 627 | 12 | 618 |
| ROSE/TRIO-012 2015 | / | / | / | / | ROSE/TRIO-012 2015 | / | / | / | / |
|  |  |  |  |  |  |  |  |  |  |
| **Total** | 593 | 3067 | 495 | 2686 | **Total** | 70 | 3067 | 80 | 2686 |
| **Fixed model (95% CI)** | 1.09 [0.95, 1.25] | | | | **Fixed model (95% CI)** | 0.78 [0.56, 1.08] | | | |
| **Heterogeneity** | Chi² = 9.64, df = 8 (P = 0.29); I² = 17% | | | | **Heterogeneity** | Chi² = 2.64, df = 7 (P = 0.92); I² = 0% | | | |
| **Test for overall effect** | Z = 1.26 (P = 0.21) | | | | **Test for overall effect** | Z = 1.50 (P = 0.13) | | | |
|  |  |  |  |  |  |  |  |  |  |
|  |  |  |  |  |  |  |  |  |  |
| **Peripheral oedema (Grade1-2)** | | | | | **Peripheral oedema (Grade>=3)** | | | | |
|  | **RAM** | | **Control** | |  | **RAM** | | **Control** | |
|  | Events | Total | Events | Total |  | Events | Total | Events | Total |
| RAINBOW 2014 | 77 | 327 | 43 | 329 | RAINBOW 2014 | 5 | 327 | 2 | 329 |
| RAINBOW-Asia 2021 | / | / | / | / | RAINBOW-Asia 2021 | / | / | / | / |
| RAINFALL 2019 | / | / | / | / | RAINFALL 2019 | / | / | / | / |
| RAISE 2015 | 107 | 529 | 48 | 528 | RAISE 2015 | 1 | 529 | 0 | 528 |
| RANGE 2020 | / | / | / | / | RANGE 2020 | / | / | / | / |
| REACH 2015 | 100 | 277 | 49 | 276 | REACH 2015 | 1 | 277 | 1 | 276 |
| REACH-2 2019 | 47 | 197 | 13 | 95 | REACH-2 2019 | 3 | 197 | 0 | 95 |
| REGARD 2014 | / | / | / | / | REGARD 2014 | / | / | / | / |
| RELAY 2019 | / | / | / | / | RELAY 2019 | / | / | / | / |
| REVEL 2014 | 102 | 627 | 53 | 618 | REVEL 2014 | 0 | 627 | 0 | 618 |
| ROSE/TRIO-012 2015 | / | / | / | / | ROSE/TRIO-012 2015 | / | / | / | / |
|  |  |  |  |  |  |  |  |  |  |
| **Total** | 433 | 1957 | 206 | 1846 | **Total** | 10 | 1957 | 3 | 1846 |
| **Fixed model (95% CI)** | 2.28 [1.90, 2.73] | | | | **Fixed model (95% CI)** | 2.37 [0.74, 7.58] | | | |
| **Heterogeneity** | Chi² = 1.52, df = 4 (P = 0.82); I² = 0% | | | | **Heterogeneity** | Chi² = 0.46, df = 3 (P = 0.93); I² = 0% | | | |
| **Test for overall effect** | Z = 8.86 (P < 0.00001) | | | | **Test for overall effect** | Z = 1.45 (P = 0.15) | | | |
|  |  |  |  |  |  |  |  |  |  |
|  |  |  |  |  |  |  |  |  |  |
| **Constipation (Grade1-2)** | | | | | **Constipation (Grade>=3)** | | | | |
|  | **RAM** | | **Control** | |  | **RAM** | | **Control** | |
|  | Events | Total | Events | Total |  | Events | Total | Events | Total |
| RAINBOW 2014 | 70 | 327 | 69 | 329 | RAINBOW 2014 | 0 | 327 | 2 | 329 |
| RAINBOW-Asia 2021 | 50 | 293 | 26 | 145 | RAINBOW-Asia 2021 | 0 | 293 | 0 | 145 |
| RAINFALL 2019 | / | / | / | / | RAINFALL 2019 | / | / | / | / |
| RAISE 2015 | 146 | 529 | 112 | 528 | RAISE 2015 | 5 | 529 | 8 | 528 |
| RANGE 2020 | 16 | 258 | 21 | 265 | RANGE 2020 | 1 | 258 | 0 | 265 |
| REACH 2015 | 36 | 277 | 34 | 276 | REACH 2015 | 0 | 277 | 0 | 276 |
| REACH-2 2019 | 26 | 197 | 18 | 95 | REACH-2 2019 | 1 | 197 | 1 | 95 |
| REGARD 2014 | 36 | 236 | 26 | 115 | REGARD 2014 | 1 | 236 | 3 | 115 |
| RELAY 2019 | / | / | / | / | RELAY 2019 | / | / | / | / |
| REVEL 2014 | 101 | 627 | 108 | 618 | REVEL 2014 | 1 | 627 | 6 | 618 |
| ROSE/TRIO-012 2015 | / | / | / | / | ROSE/TRIO-012 2015 | / | / | / | / |
|  |  |  |  |  |  |  |  |  |  |
| **Total** | 481 | 2744 | 414 | 2371 | **Total** | 9 | 2744 | 20 | 2371 |
| **Fixed model (95% CI)** | 1.01 [0.87, 1.17] | | | | **Fixed model (95% CI)** | 0.41 [0.19, 0.88] | | | |
| **Heterogeneity** | Chi² = 11.42, df = 7 (P = 0.12); I² = 39% | | | | **Heterogeneity** | Chi² = 3.67, df = 5 (P = 0.60); I² = 0% | | | |
| **Test for overall effect** | Z = 0.14 (P = 0.89) | | | | **Test for overall effect** | Z = 2.29 (P = 0.02) | | | |
|  |  |  |  |  |  |  |  |  |  |
|  |  |  |  |  |  |  |  |  |  |
| **Stomatitis (Grade1-2)** | | | | | **Stomatitis (Grade>=3)** | | | | |
|  | **RAM** | | **Control** | |  | **RAM** | | **Control** | |
|  | Events | Total | Events | Total |  | Events | Total | Events | Total |
| RAINBOW 2014 | 62 | 327 | 22 | 329 | RAINBOW 2014 | 2 | 327 | 2 | 329 |
| RAINBOW-Asia 2021 | / | / | / | / | RAINBOW-Asia 2021 | / | / | / | / |
| RAINFALL 2019 | / | / | / | / | RAINFALL 2019 | / | / | / | / |
| RAISE 2015 | 143 | 529 | 98 | 528 | RAISE 2015 | 20 | 529 | 12 | 528 |
| RANGE 2020 | 51 | 258 | 24 | 265 | RANGE 2020 | 9 | 258 | 0 | 265 |
| REACH 2015 | / | / | / | / | REACH 2015 | / | / | / | / |
| REACH-2 2019 | / | / | / | / | REACH-2 2019 | / | / | / | / |
| REGARD 2014 | / | / | / | / | REGARD 2014 | / | / | / | / |
| RELAY 2019 | 88 | 221 | 79 | 225 | RELAY 2019 | 4 | 221 | 3 | 225 |
| REVEL 2014 | 146 | 627 | 80 | 618 | REVEL 2014 | 27 | 627 | 10 | 618 |
| ROSE/TRIO-012 2015 | 381 | 752 | 117 | 382 | ROSE/TRIO-012 2015 | 46 | 752 | 4 | 382 |
|  |  |  |  |  |  |  |  |  |  |
| **Total** | 871 | 2714 | 420 | 2347 | **Total** | 108 | 2714 | 31 | 2347 |
| **Random model (95% CI)** | 2.00 [1.57, 2.54] | | | | **Fixed model (95% CI)** | 2.92 [1.94, 4.40] | | | |
| **Heterogeneity** | Tau² = 0.05; Chi² = 13.68, df = 5 (P = 0.02); I² = 63% | | | | **Heterogeneity** | Chi² = 8.11, df = 5 (P = 0.15); I² = 38% | | | |
| **Test for overall effect** | Z = 5.66 (P < 0.00001) | | | | **Test for overall effect** | Z = 5.14 (P < 0.00001) | | | |
|  |  |  |  |  |  |  |  |  |  |
|  |  |  |  |  |  |  |  |  |  |
| **Pyrexia (Grade1-2)** | | | | | **Pyrexia (Grade>=3)** | | | | |
|  | **RAM** | | **Control** | |  | **RAM** | | **Control** | |
|  | Events | Total | Events | Total |  | Events | Total | Events | Total |
| RAINBOW 2014 | 56 | 327 | 36 | 329 | RAINBOW 2014 | 3 | 327 | 1 | 329 |
| RAINBOW-Asia 2021 | 55 | 293 | 24 | 145 | RAINBOW-Asia 2021 | 1 | 293 | 0 | 145 |
| RAINFALL 2019 | / | / | / | / | RAINFALL 2019 | / | / | / | / |
| RAISE 2015 | 78 | 529 | 55 | 528 | RAISE 2015 | 2 | 529 | 1 | 528 |
| RANGE 2020 | 22 | 258 | 15 | 265 | RANGE 2020 | 1 | 258 | 1 | 265 |
| REACH 2015 | 45 | 277 | 25 | 276 | REACH 2015 | 1 | 277 | 1 | 276 |
| REACH-2 2019 | 20 | 197 | 3 | 95 | REACH-2 2019 | 0 | 197 | 0 | 95 |
| REGARD 2014 | / | / | / | / | REGARD 2014 | / | / | / | / |
| RELAY 2019 | 47 | 221 | 27 | 225 | RELAY 2019 | 0 | 221 | 1 | 225 |
| REVEL 2014 | 104 | 627 | 80 | 618 | REVEL 2014 | 3 | 627 | 2 | 618 |
| ROSE/TRIO-012 2015 | / | / | / | / | ROSE/TRIO-012 2015 | / | / | / | / |
|  |  |  |  |  |  |  |  |  |  |
| **Total** | 427 | 2729 | 265 | 2481 | **Total** | 11 | 2729 | 7 | 2481 |
| **Fixed model (95% CI)** | 1.55 [1.32, 1.83] | | | | **Fixed model (95% CI)** | 1.41 [0.57, 3.48] | | | |
| **Heterogeneity** | Chi² = 5.37, df = 7 (P = 0.61); I² = 0% | | | | **Heterogeneity** | Chi² = 1.40, df = 6 (P = 0.97); I² = 0% | | | |
| **Test for overall effect** | Z = 5.22 (P < 0.00001) | | | | **Test for overall effect** | Z = 0.75 (P = 0.45) | | | |
|  |  |  |  |  |  |  |  |  |  |
|  |  |  |  |  |  |  |  |  |  |
| **Proteinuria (Grade1-2)** | | | | | **Proteinuria (Grade>=3)** | | | | |
|  | **RAM** | | **Control** | |  | **RAM** | | **Control** | |
|  | Events | Total | Events | Total |  | Events | Total | Events | Total |
| RAINBOW 2014 | 50 | 327 | 20 | 329 | RAINBOW 2014 | 4 | 327 | 0 | 329 |
| RAINBOW-Asia 2021 | 92 | 293 | 30 | 145 | RAINBOW-Asia 2021 | 6 | 293 | 1 | 145 |
| RAINFALL 2019 | / | / | / | / | RAINFALL 2019 | / | / | / | / |
| RAISE 2015 | / | / | / | / | RAISE 2015 | / | / | / | / |
| RANGE 2020 | 20 | 258 | 8 | 265 | RANGE 2020 | 2 | 258 | 1 | 265 |
| REACH 2015 | 39 | 277 | 13 | 276 | REACH 2015 | 6 | 277 | 0 | 276 |
| REACH-2 2019 | / | / | / | / | REACH-2 2019 | / | / | / | / |
| REGARD 2014 | / | / | / | / | REGARD 2014 | / | / | / | / |
| RELAY 2019 | 69 | 221 | 19 | 225 | RELAY 2019 | 6 | 221 | 0 | 225 |
| REVEL 2014 | / | / | / | / | REVEL 2014 | / | / | / | / |
| ROSE/TRIO-012 2015 | / | / | / | / | ROSE/TRIO-012 2015 | / | / | / | / |
|  |  |  |  |  |  |  |  |  |  |
| **Total** | 270 | 1376 | 90 | 1240 | **Total** | 24 | 1376 | 2 | 1240 |
| **Random model (95% CI)** | 2.89 [1.98, 4.21] | | | | **Fixed model (95% CI)** | 6.26 [2.05, 19.10] | | | |
| **Heterogeneity** | Tau² = 0.09; Chi² = 8.09, df = 4 (P = 0.09); I² = 51% | | | | **Heterogeneity** | Chi² = 1.88, df = 4 (P = 0.76); I² = 0% | | | |
| **Test for overall effect** | Z = 5.52 (P < 0.00001) | | | | **Test for overall effect** | Z = 3.22 (P = 0.001) | | | |
|  |  |  |  |  |  |  |  |  |  |
|  |  |  |  |  |  |  |  |  |  |
| **Dyspnoea (Grade1-2)** | | | | | **Dyspnoea (Grade>=3)** | | | | |
|  | **RAM** | | **Control** | |  | **RAM** | | **Control** | |
|  | Events | Total | Events | Total |  | Events | Total | Events | Total |
| RAINBOW 2014 | 34 | 327 | 29 | 329 | RAINBOW 2014 | 8 | 327 | 2 | 329 |
| RAINBOW-Asia 2021 | / | / | / | / | RAINBOW-Asia 2021 | / | / | / | / |
| RAINFALL 2019 | / | / | / | / | RAINFALL 2019 | / | / | / | / |
| RAISE 2015 | 49 | 529 | 42 | 528 | RAISE 2015 | 4 | 529 | 6 | 528 |
| RANGE 2020 | 7 | 258 | 10 | 265 | RANGE 2020 | 4 | 258 | 1 | 265 |
| REACH 2015 | / | / | / | / | REACH 2015 | / | / | / | / |
| REACH-2 2019 | 1 | 197 | 0 | 95 | REACH-2 2019 | 0 | 197 | 0 | 95 |
| REGARD 2014 | 22 | 236 | 15 | 115 | REGARD 2014 | 4 | 236 | 7 | 115 |
| RELAY 2019 | / | / | / | / | RELAY 2019 | / | / | / | / |
| REVEL 2014 | 138 | 627 | 149 | 618 | REVEL 2014 | 24 | 627 | 51 | 618 |
| ROSE/TRIO-012 2015 | / | / | / | / | ROSE/TRIO-012 2015 | / | / | / | / |
|  |  |  |  |  |  |  |  |  |  |
| **Total** | 251 | 2174 | 245 | 1950 | **Total** | 44 | 2174 | 67 | 1950 |
| **Fixed model (95% CI)** | 0.96 [0.79, 1.16] | | | | **Random model (95% CI)** | 0.82 [0.32, 2.09] | | | |
| **Heterogeneity** | Chi² = 3.25, df = 5 (P = 0.66); I² = 0% | | | | **Heterogeneity** | Tau² = 0.69; Chi² = 11.89, df = 4 (P = 0.02); I² = 66% | | | |
| **Test for overall effect** | Z = 0.45 (P = 0.65) | | | | **Test for overall effect** | Z = 0.41 (P = 0.68) | | | |
|  |  |  |  |  |  |  |  |  |  |
|  |  |  |  |  |  |  |  |  |  |
| **Rash (Grade1-2)** | | | | | **Rash (Grade>=3)** | | | | |
|  | **RAM** | | **Control** | |  | **RAM** | | **Control** | |
|  | Events | Total | Events | Total |  | Events | Total | Events | Total |
| RAINBOW 2014 | 42 | 327 | 31 | 329 | RAINBOW 2014 | 0 | 327 | 0 | 329 |
| RAINBOW-Asia 2021 | / | / | / | / | RAINBOW-Asia 2021 | / | / | / | / |
| RAINFALL 2019 | / | / | / | / | RAINFALL 2019 | / | / | / | / |
| RAISE 2015 | 57 | 529 | 50 | 528 | RAISE 2015 | 0 | 529 | 3 | 528 |
| RANGE 2020 | 1 | 258 | 1 | 265 | RANGE 2020 | 1 | 258 | 0 | 265 |
| REACH 2015 | / | / | / | / | REACH 2015 | / | / | / | / |
| REACH-2 2019 | / | / | / | / | REACH-2 2019 | / | / | / | / |
| REGARD 2014 | / | / | / | / | REGARD 2014 | / | / | / | / |
| RELAY 2019 | 37 | 221 | 49 | 225 | RELAY 2019 | 2 | 221 | 5 | 225 |
| REVEL 2014 | / | / | / | / | REVEL 2014 | / | / | / | / |
| ROSE/TRIO-012 2015 | / | / | / | / | ROSE/TRIO-012 2015 | / | / | / | / |
|  |  |  |  |  |  |  |  |  |  |
| **Total** | 137 | 1335 | 131 | 1347 | **Total** | 3 | 1335 | 8 | 1347 |
| **Fixed model (95% CI)** | 1.06 [0.82, 1.37] | | | | **Fixed model (95% CI)** | 0.45 [0.14, 1.46] | | | |
| **Heterogeneity** | Chi² = 4.02, df = 3 (P = 0.26); I² = 25% | | | | **Heterogeneity** | Chi² = 1.99, df = 2 (P = 0.37); I² = 0% | | | |
| **Test for overall effect** | Z = 0.45 (P = 0.65) | | | | **Test for overall effect** | Z = 1.33 (P = 0.18) | | | |
|  |  |  |  |  |  |  |  |  |  |
|  |  |  |  |  |  |  |  |  |  |
| **Weight decreased (Grade1-2)** | | | | | **Weight decreased (Grade>=3)** | | | | |
|  | **RAM** | | **Control** | |  | **RAM** | | **Control** | |
|  | Events | Total | Events | Total |  | Events | Total | Events | Total |
| RAINBOW 2014 | 39 | 327 | 45 | 329 | RAINBOW 2014 | 6 | 327 | 4 | 329 |
| RAINBOW-Asia 2021 | 58 | 293 | 21 | 145 | RAINBOW-Asia 2021 | 0 | 293 | 1 | 145 |
| RAINFALL 2019 | / | / | / | / | RAINFALL 2019 | / | / | / | / |
| RAISE 2015 | 67 | 529 | 40 | 528 | RAISE 2015 | 2 | 529 | 0 | 528 |
| RANGE 2020 | / | / | / | / | RANGE 2020 | / | / | / | / |
| REACH 2015 | / | / | / | / | REACH 2015 | / | / | / | / |
| REACH-2 2019 | / | / | / | / | REACH-2 2019 | / | / | / | / |
| REGARD 2014 | / | / | / | / | REGARD 2014 | / | / | / | / |
| RELAY 2019 | / | / | / | / | RELAY 2019 | / | / | / | / |
| REVEL 2014 | / | / | / | / | REVEL 2014 | / | / | / | / |
| ROSE/TRIO-012 2015 | / | / | / | / | ROSE/TRIO-012 2015 | / | / | / | / |
|  |  |  |  |  |  |  |  |  |  |
| **Total** | 164 | 1149 | 106 | 1002 | **Total** | 8 | 1149 | 5 | 1002 |
| **Random model (95% CI)** | 1.31 [0.83, 2.05] | | | | **Fixed model (95% CI)** | 1.37 [0.49, 3.80] | | | |
| **Heterogeneity** | Tau² = 0.10; Chi² = 5.52, df = 2 (P = 0.06); I² = 64% | | | | **Heterogeneity** | Chi² = 2.41, df = 2 (P = 0.30); I² = 17% | | | |
| **Test for overall effect** | Z = 1.16 (P = 0.25) | | | | **Test for overall effect** | Z = 0.60 (P = 0.55) | | | |
|  |  |  |  |  |  |  |  |  |  |
|  |  |  |  |  |  |  |  |  |  |
| **Malignant neoplasm progression (Grade1-2)** | | | | | **Malignant neoplasm progression (Grade>=3)** | | | | |
|  | **RAM** | | **Control** | |  | **RAM** | | **Control** | |
|  | Events | Total | Events | Total |  | Events | Total | Events | Total |
| RAINBOW 2014 | 5 | 327 | 1 | 329 | RAINBOW 2014 | 47 | 327 | 59 | 329 |
| RAINBOW-Asia 2021 | / | / | / | / | RAINBOW-Asia 2021 | / | / | / | / |
| RAINFALL 2019 | / | / | / | / | RAINFALL 2019 | / | / | / | / |
| RAISE 2015 | / | / | / | / | RAISE 2015 | / | / | / | / |
| RANGE 2020 | / | / | / | / | RANGE 2020 | / | / | / | / |
| REACH 2015 | 0 | 277 | 0 | 276 | REACH 2015 | 18 | 277 | 11 | 276 |
| REACH-2 2019 | / | / | / | / | REACH-2 2019 | / | / | / | / |
| REGARD 2014 | / | / | / | / | REGARD 2014 | / | / | / | / |
| RELAY 2019 | / | / | / | / | RELAY 2019 | / | / | / | / |
| REVEL 2014 | / | / | / | / | REVEL 2014 | / | / | / | / |
| ROSE/TRIO-012 2015 | / | / | / | / | ROSE/TRIO-012 2015 | / | / | / | / |
|  |  |  |  |  |  |  |  |  |  |
| **Total** | 5 | 604 | 1 | 605 | **Total** | 65 | 604 | 70 | 605 |
| **Fixed model (95% CI)** | 5.09 [0.59, 43.83] | | | | **Random model (95% CI)** | 1.06 [0.50, 2.24] | | | |
| **Heterogeneity** | Not applicable | | | | **Heterogeneity** | Tau² = 0.20; Chi² = 3.04, df = 1 (P = 0.08); I² = 67% | | | |
| **Test for overall effect** | Z = 1.48 (P = 0.14) | | | | **Test for overall effect** | Z = 0.15 (P = 0.88) | | | |
|  |  |  |  |  |  |  |  |  |  |
|  |  |  |  |  |  |  |  |  |  |
| **Cough (Grade1-2)** | | | | | **Cough (Grade>=3)** | | | | |
|  | **RAM** | | **Control** | |  | **RAM** | | **Control** | |
|  | Events | Total | Events | Total |  | Events | Total | Events | Total |
| RAINBOW 2014 | 40 | 327 | 25 | 329 | RAINBOW 2014 | 0 | 327 | 0 | 329 |
| RAINBOW-Asia 2021 | 30 | 293 | 9 | 145 | RAINBOW-Asia 2021 | 0 | 293 | 0 | 145 |
| RAINFALL 2019 | / | / | / | / | RAINFALL 2019 | / | / | / | / |
| RAISE 2015 | 66 | 529 | 40 | 528 | RAISE 2015 | 0 | 529 | 2 | 528 |
| RANGE 2020 | / | / | / | / | RANGE 2020 | 0 | 0 | 0 | 0 |
| REACH 2015 | 40 | 277 | 24 | 276 | REACH 2015 | 1 | 277 | 0 | 276 |
| REACH-2 2019 | / | / | / | / | REACH-2 2019 | / | / | / | / |
| REGARD 2014 | / | / | / | / | REGARD 2014 | / | / | / | / |
| RELAY 2019 | 47 | 221 | 35 | 225 | RELAY 2019 | 1 | 221 | 0 | 225 |
| REVEL 2014 | 133 | 627 | 128 | 618 | REVEL 2014 | 3 | 627 | 5 | 618 |
| ROSE/TRIO-012 2015 | / | / | / | / | ROSE/TRIO-012 2015 | / | / | / | / |
|  |  |  |  |  |  |  |  |  |  |
| **Total** | 356 | 2274 | 261 | 2121 | **Total** | 5 | 2274 | 7 | 2121 |
| **Fixed model (95% CI)** | 1.37 [1.15, 1.63] | | | | **Fixed model (95% CI)** | 0.76 [0.27, 2.11] | | | |
| **Heterogeneity** | Chi² = 7.39, df = 5 (P = 0.19); I² = 32% | | | | **Heterogeneity** | Chi² = 2.30, df = 3 (P = 0.51); I² = 0% | | | |
| **Test for overall effect** | Z = 3.54 (P = 0.0004) | | | | **Test for overall effect** | Z = 0.53 (P = 0.60) | | | |
|  |  |  |  |  |  |  |  |  |  |
|  |  |  |  |  |  |  |  |  |  |
| **Back pain (Grade1-2)** | | | | | **Back pain (Grade>=3)** | | | | |
|  | **RAM** | | **Control** | |  | **RAM** | | **Control** | |
|  | Events | Total | Events | Total |  | Events | Total | Events | Total |
| RAINBOW 2014 | 35 | 327 | 35 | 329 | RAINBOW 2014 | 4 | 327 | 5 | 329 |
| RAINBOW-Asia 2021 | 32 | 293 | 14 | 145 | RAINBOW-Asia 2021 | 0 | 293 | 0 | 145 |
| RAINFALL 2019 | / | / | / | / | RAINFALL 2019 | / | / | / | / |
| RAISE 2015 | / | / | / | / | RAISE 2015 | / | / | / | / |
| RANGE 2020 | 5 | 258 | 2 | 265 | RANGE 2020 | 1 | 258 | 0 | 265 |
| REACH 2015 | / | / | / | / | REACH 2015 | / | / | / | / |
| REACH-2 2019 | / | / | / | / | REACH-2 2019 | / | / | / | / |
| REGARD 2014 | / | / | / | / | REGARD 2014 | / | / | / | / |
| RELAY 2019 | / | / | / | / | RELAY 2019 | / | / | / | / |
| REVEL 2014 | 71 | 627 | 53 | 618 | REVEL 2014 | 7 | 627 | 2 | 618 |
| ROSE/TRIO-012 2015 | / | / | / | / | ROSE/TRIO-012 2015 | / | / | / | / |
|  |  |  |  |  |  |  |  |  |  |
| **Total** | 143 | 1505 | 104 | 1357 | **Total** | 12 | 1505 | 7 | 1357 |
| **Fixed model (95% CI)** | 1.24 [0.95, 1.62] | | | | **Fixed model (95% CI)** | 1.67 [0.67, 4.16] | | | |
| **Heterogeneity** | Chi² = 1.74, df = 3 (P = 0.63); I² = 0% | | | | **Heterogeneity** | Chi² = 2.15, df = 2 (P = 0.34); I² = 7% | | | |
| **Test for overall effect** | Z = 1.55 (P = 0.12) | | | | **Test for overall effect** | Z = 1.11 (P = 0.27) | | | |
|  |  |  |  |  |  |  |  |  |  |
|  |  |  |  |  |  |  |  |  |  |
| **Hypoalbuminaemia (Grade1-2)** | | | | | **Hypoalbuminaemia (Grade>=3)** | | | | |
|  | **RAM** | | **Control** | |  | **RAM** | | **Control** | |
|  | Events | Total | Events | Total |  | Events | Total | Events | Total |
| RAINBOW 2014 | 32 | 327 | 13 | 329 | RAINBOW 2014 | 4 | 327 | 3 | 329 |
| RAINBOW-Asia 2021 | 88 | 293 | 30 | 145 | RAINBOW-Asia 2021 | 1 | 293 | 1 | 145 |
| RAINFALL 2019 | / | / | / | / | RAINFALL 2019 | / | / | / | / |
| RAISE 2015 | / | / | / | / | RAISE 2015 | / | / | / | / |
| RANGE 2020 | / | / | / | / | RANGE 2020 | / | / | / | / |
| REACH 2015 | 30 | 277 | 13 | 276 | REACH 2015 | 3 | 277 | 0 | 276 |
| REACH-2 2019 | / | / | / | / | REACH-2 2019 | / | / | / | / |
| REGARD 2014 | / | / | / | / | REGARD 2014 | / | / | / | / |
| RELAY 2019 | / | / | / | / | RELAY 2019 | / | / | / | / |
| REVEL 2014 | / | / | / | / | REVEL 2014 | / | / | / | / |
| ROSE/TRIO-012 2015 | / | / | / | / | ROSE/TRIO-012 2015 | / | / | / | / |
|  |  |  |  |  |  |  |  |  |  |
| **Total** | 150 | 897 | 56 | 750 | **Total** | 8 | 897 | 4 | 750 |
| **Fixed model (95% CI)** | 2.05 [1.47, 2.87] | | | | **Fixed model (95% CI)** | 1.70 [0.54, 5.30] | | | |
| **Heterogeneity** | Chi² = 1.66, df = 2 (P = 0.44); I² = 0% | | | | **Heterogeneity** | Chi² = 1.74, df = 2 (P = 0.42); I² = 0% | | | |
| **Test for overall effect** | Z = 4.23 (P < 0.0001) | | | | **Test for overall effect** | Z = 0.91 (P = 0.36) | | | |
|  |  |  |  |  |  |  |  |  |  |
|  |  |  |  |  |  |  |  |  |  |
| **Myalgia (Grade1-2)** | | | | | **Myalgia (Grade>=3)** | | | | |
|  | **RAM** | | **Control** | |  | **RAM** | | **Control** | |
|  | Events | Total | Events | Total |  | Events | Total | Events | Total |
| RAINBOW 2014 | 34 | 327 | 32 | 329 | RAINBOW 2014 | 0 | 327 | 1 | 329 |
| RAINBOW-Asia 2021 | / | / | / | / | RAINBOW-Asia 2021 | / | / | / | / |
| RAINFALL 2019 | / | / | / | / | RAINFALL 2019 | / | / | / | / |
| RAISE 2015 | / | / | / | / | RAISE 2015 | / | / | / | / |
| RANGE 2020 | / | / | / | / | RANGE 2020 | / | / | / | / |
| REACH 2015 | / | / | / | / | REACH 2015 | / | / | / | / |
| REACH-2 2019 | / | / | / | / | REACH-2 2019 | / | / | / | / |
| REGARD 2014 | / | / | / | / | REGARD 2014 | / | / | / | / |
| RELAY 2019 | / | / | / | / | RELAY 2019 | / | / | / | / |
| REVEL 2014 | 78 | 627 | 44 | 618 | REVEL 2014 | 4 | 627 | 4 | 618 |
| ROSE/TRIO-012 2015 | / | / | / | / | ROSE/TRIO-012 2015 | / | / | / | / |
|  |  |  |  |  |  |  |  |  |  |
| **Total** | 112 | 954 | 76 | 947 | **Total** | 4 | 954 | 5 | 947 |
| **Random model (95% CI)** | 1.52 [1.12, 2.07] | | | | **Fixed model (95% CI)** | 0.81 [0.23, 2.82] | | | |
| **Heterogeneity** | Chi² = 2.77, df = 1 (P = 0.10); I² = 64% | | | | **Heterogeneity** | Chi² = 0.37, df = 1 (P = 0.54); I² = 0% | | | |
| **Test for overall effect** | Z = 2.70 (P = 0.007) | | | | **Test for overall effect** | Z = 0.33 (P = 0.74) | | | |
|  |  |  |  |  |  |  |  |  |  |
|  |  |  |  |  |  |  |  |  |  |
| **Ascites (Grade1-2)** | | | | | **Ascites (Grade>=3)** | | | | |
|  | **RAM** | | **Control** | |  | **RAM** | | **Control** | |
|  | Events | Total | Events | Total |  | Events | Total | Events | Total |
| RAINBOW 2014 | 21 | 327 | 14 | 329 | RAINBOW 2014 | 12 | 327 | 13 | 329 |
| RAINBOW-Asia 2021 | / | / | / | / | RAINBOW-Asia 2021 | / | / | / | / |
| RAINFALL 2019 | / | / | / | / | RAINFALL 2019 | / | / | / | / |
| RAISE 2015 | / | / | / | / | RAISE 2015 | / | / | / | / |
| RANGE 2020 | / | / | / | / | RANGE 2020 | / | / | / | / |
| REACH 2015 | 61 | 277 | 29 | 276 | REACH 2015 | 13 | 277 | 11 | 276 |
| REACH-2 2019 | 27 | 197 | 5 | 95 | REACH-2 2019 | 8 | 197 | 2 | 95 |
| REGARD 2014 | / | / | / | / | REGARD 2014 | / | / | / | / |
| RELAY 2019 | / | / | / | / | RELAY 2019 | / | / | / | / |
| REVEL 2014 | / | / | / | / | REVEL 2014 | / | / | / | / |
| ROSE/TRIO-012 2015 | / | / | / | / | ROSE/TRIO-012 2015 | / | / | / | / |
|  |  |  |  |  |  |  |  |  |  |
| **Total** | 109 | 801 | 48 | 700 | **Total** | 33 | 801 | 26 | 700 |
| **Fixed model (95% CI)** | 2.20 [1.53, 3.16] | | | | **Fixed model (95% CI)** | 1.14 [0.67, 1.94] | | | |
| **Heterogeneity** | Chi² = 1.40, df = 2 (P = 0.50); I² = 0% | | | | **Heterogeneity** | Chi² = 0.73, df = 2 (P = 0.69); I² = 0% | | | |
| **Test for overall effect** | Z = 4.24 (P < 0.0001) | | | | **Test for overall effect** | Z = 0.48 (P = 0.63) | | | |
|  |  |  |  |  |  |  |  |  |  |
|  |  |  |  |  |  |  |  |  |  |
| **Headache (Grade1-2)** | | | | | **Headache (Grade>=3)** | | | | |
|  | **RAM** | | **Control** | |  | **RAM** | | **Control** | |
|  | Events | Total | Events | Total |  | Events | Total | Events | Total |
| RAINBOW 2014 | 32 | 327 | 21 | 329 | RAINBOW 2014 | 0 | 327 | 1 | 329 |
| RAINBOW-Asia 2021 | / | / | / | / | RAINBOW-Asia 2021 | / | / | / | / |
| RAINFALL 2019 | / | / | / | / | RAINFALL 2019 | / | / | / | / |
| RAISE 2015 | 75 | 529 | 41 | 528 | RAISE 2015 | 3 | 529 | 0 | 528 |
| RANGE 2020 | / | / | / | / | RANGE 2020 | 0 | 0 | 0 | 0 |
| REACH 2015 | 51 | 277 | 15 | 276 | REACH 2015 | 2 | 277 | 0 | 276 |
| REACH-2 2019 | 28 | 197 | 4 | 95 | REACH-2 2019 | 0 | 197 | 1 | 95 |
| REGARD 2014 | / | / | / | / | REGARD 2014 | / | / | / | / |
| RELAY 2019 | / | / | / | / | RELAY 2019 | / | / | / | / |
| REVEL 2014 | 66 | 627 | 67 | 618 | REVEL 2014 | 3 | 627 | 6 | 618 |
| ROSE/TRIO-012 2015 | / | / | / | / | ROSE/TRIO-012 2015 | / | / | / | / |
|  |  |  |  |  |  |  |  |  |  |
| **Total** | 252 | 1957 | 148 | 1846 | **Total** | 8 | 1957 | 8 | 1846 |
| **Random model (95% CI)** | 1.98 [1.17, 3.35] | | | | **Fixed model (95% CI)** | 0.93 [0.39, 2.22] | | | |
| **Heterogeneity** | Tau² = 0.27; Chi² = 19.48, df = 4 (P = 0.0006); I² = 79% | | | | **Heterogeneity** | Chi² = 5.33, df = 4 (P = 0.26); I² = 25% | | | |
| **Test for overall effect** | Z = 2.54 (P = 0.01) | | | | **Test for overall effect** | Z = 0.17 (P = 0.87) | | | |
|  |  |  |  |  |  |  |  |  |  |
|  |  |  |  |  |  |  |  |  |  |
| **Neutropenia (Grade1-2)** | | | | | **Neutropenia (Grade>=3)** | | | | |
|  | **RAM** | | **Control** | |  | **RAM** | | **Control** | |
|  | Events | Total | Events | Total |  | Events | Total | Events | Total |
| RAINBOW 2014 | 45 | 327 | 40 | 329 | RAINBOW 2014 | 133 | 327 | 62 | 329 |
| RAINBOW-Asia 2021 | / | / | / | / | RAINBOW-Asia 2021 | / | / | / | / |
| RAINFALL 2019 | 89 | 323 | 83 | 315 | RAINFALL 2019 | 85 | 323 | 85 | 315 |
| RAISE 2015 | 108 | 529 | 118 | 528 | RAISE 2015 | 203 | 529 | 123 | 528 |
| RANGE 2020 | 5 | 258 | 5 | 265 | RANGE 2020 | 17 | 258 | 6 | 265 |
| REACH 2015 | / | / | / | / | REACH 2015 | / | / | / | / |
| REACH-2 2019 | / | / | / | / | REACH-2 2019 | / | / | / | / |
| REGARD 2014 | / | / | / | / | REGARD 2014 | / | / | / | / |
| RELAY 2019 | / | / | / | / | RELAY 2019 | / | / | / | / |
| REVEL 2014 | 345 | 627 | 284 | 618 | REVEL 2014 | 306 | 627 | 246 | 618 |
| ROSE/TRIO-012 2015 | 132 | 752 | 61 | 382 | ROSE/TRIO-012 2015 | 114 | 752 | 50 | 382 |
|  |  |  |  |  |  |  |  |  |  |
| **Total** | 724 | 2816 | 591 | 2437 | **Total** | 858 | 2816 | 572 | 2437 |
| **Fixed model (95% CI)** | 1.16 [1.02, 1.33] | | | | **Random model (95% CI)** | 1.67 [1.20, 2.33] | | | |
| **Heterogeneity** | Chi² = 6.97, df = 5 (P = 0.22); I² = 28% | | | | **Heterogeneity** | Tau² = 0.13; Chi² = 28.03, df = 5 (P < 0.0001); I² = 82% | | | |
| **Test for overall effect** | Z = 2.19 (P = 0.03) | | | | **Test for overall effect** | Z = 3.02 (P = 0.002) | | | |
|  |  |  |  |  |  |  |  |  |  |
|  |  |  |  |  |  |  |  |  |  |
| **Anaemia (Grade1-2)** | | | | | **Anaemia (Grade>=3)** | | | | |
|  | **RAM** | | **Control** | |  | **RAM** | | **Control** | |
|  | Events | Total | Events | Total |  | Events | Total | Events | Total |
| RAINBOW 2014 | 84 | 327 | 85 | 329 | RAINBOW 2014 | 30 | 327 | 34 | 329 |
| RAINBOW-Asia 2021 | / | / | / | / | RAINBOW-Asia 2021 | 46 | 293 | 24 | 145 |
| RAINFALL 2019 | 71 | 323 | 73 | 315 | RAINFALL 2019 | 39 | 323 | 44 | 315 |
| RAISE 2015 | 78 | 529 | 91 | 528 | RAISE 2015 | 8 | 529 | 19 | 528 |
| RANGE 2020 | 25 | 258 | 29 | 265 | RANGE 2020 | 5 | 258 | 14 | 265 |
| REACH 2015 | / | / | / | / | REACH 2015 | / | / | / | / |
| REACH-2 2019 | / | / | / | / | REACH-2 2019 | / | / | / | / |
| REGARD 2014 | 35 | 236 | 17 | 115 | REGARD 2014 | 15 | 236 | 9 | 115 |
| RELAY 2019 | / | / | / | / | RELAY 2019 | / | / | / | / |
| REVEL 2014 | 131 | 627 | 174 | 618 | REVEL 2014 | 18 | 627 | 35 | 618 |
| ROSE/TRIO-012 2015 | / | / | / | / | ROSE/TRIO-012 2015 | / | / | / | / |
|  |  |  |  |  |  |  |  |  |  |
| **Total** | 424 | 2300 | 469 | 2170 | **Total** | 161 | 2593 | 179 | 2315 |
| **Fixed model (95% CI)** | 0.83 [0.71, 0.96] | | | | **Fixed model (95% CI)** | 0.71 [0.56, 0.89] | | | |
| **Heterogeneity** | Chi² = 4.23, df = 5 (P = 0.52); I² = 0% | | | | **Heterogeneity** | Chi² = 7.20, df = 6 (P = 0.30); I² = 17% | | | |
| **Test for overall effect** | Z = 2.49 (P = 0.01) | | | | **Test for overall effect** | Z = 2.95 (P = 0.003) | | | |
|  |  |  |  |  |  |  |  |  |  |
|  |  |  |  |  |  |  |  |  |  |
| **Leucopenia (Grade1-2)** | | | | | **Leucopenia (Grade>=3)** | | | | |
|  | **RAM** | | **Control** | |  | **RAM** | | **Control** | |
|  | Events | Total | Events | Total |  | Events | Total | Events | Total |
| RAINBOW 2014 | 54 | 327 | 47 | 329 | RAINBOW 2014 | 57 | 327 | 22 | 329 |
| RAINBOW-Asia 2021 | / | / | / | / | RAINBOW-Asia 2021 | / | / | / | / |
| RAINFALL 2019 | 30 | 323 | 20 | 315 | RAINFALL 2019 | 16 | 323 | 17 | 315 |
| RAISE 2015 | / | / | / | / | RAISE 2015 | / | / | / | / |
| RANGE 2020 | / | / | / | / | RANGE 2020 | / | / | / | / |
| REACH 2015 | / | / | / | / | REACH 2015 | / | / | / | / |
| REACH-2 2019 | / | / | / | / | REACH-2 2019 | / | / | / | / |
| REGARD 2014 | / | / | / | / | REGARD 2014 | / | / | / | / |
| RELAY 2019 | / | / | / | / | RELAY 2019 | / | / | / | / |
| REVEL 2014 | 134 | 627 | 86 | 618 | REVEL 2014 | 117 | 627 | 77 | 618 |
| ROSE/TRIO-012 2015 | / | / | / | / | ROSE/TRIO-012 2015 | / | / | / | / |
|  |  |  |  |  |  |  |  |  |  |
| **Total** | 218 | 1277 | 153 | 1262 | **Total** | 190 | 1277 | 116 | 1262 |
| **Fixed model (95% CI)** | 1.50 [1.20, 1.88] | | | | **Random model (95% CI)** | 1.69 [0.97, 2.94] | | | |
| **Heterogeneity** | Chi² = 1.73, df = 2 (P = 0.42); I² = 0% | | | | **Heterogeneity** | Tau² = 0.17; Chi² = 7.44, df = 2 (P = 0.02); I² = 73% | | | |
| **Test for overall effect** | Z = 3.55 (P = 0.0004) | | | | **Test for overall effect** | Z = 1.87 (P = 0.06) | | | |
|  |  |  |  |  |  |  |  |  |  |
|  |  |  |  |  |  |  |  |  |  |
| **Thrombocytopenia (Grade1-2)** | | | | | **Thrombocytopenia (Grade>=3)** | | | | |
|  | **RAM** | | **Control** | |  | **RAM** | | **Control** | |
|  | Events | Total | Events | Total |  | Events | Total | Events | Total |
| RAINBOW 2014 | 38 | 327 | 14 | 329 | RAINBOW 2014 | 5 | 327 | 6 | 329 |
| RAINBOW-Asia 2021 | / | / | / | / | RAINBOW-Asia 2021 | / | / | / | / |
| RAINFALL 2019 | 86 | 323 | 50 | 315 | RAINFALL 2019 | 25 | 323 | 11 | 315 |
| RAISE 2015 | 134 | 529 | 68 | 528 | RAISE 2015 | 16 | 529 | 4 | 528 |
| RANGE 2020 | 4 | 258 | 0 | 265 | RANGE 2020 | 1 | 258 | 0 | 265 |
| REACH 2015 | 35 | 277 | 11 | 276 | REACH 2015 | 13 | 277 | 1 | 276 |
| REACH-2 2019 | / | / | / | / | REACH-2 2019 | / | / | / | / |
| REGARD 2014 | / | / | / | / | REGARD 2014 | / | / | / | / |
| RELAY 2019 | / | / | / | / | RELAY 2019 | / | / | / | / |
| REVEL 2014 | 84 | 627 | 32 | 618 | REVEL 2014 | 18 | 627 | 4 | 618 |
| ROSE/TRIO-012 2015 | / | / | / | / | ROSE/TRIO-012 2015 | / | / | / | / |
|  |  |  |  |  |  |  |  |  |  |
| **Total** | 381 | 2341 | 175 | 2331 | **Total** | 78 | 2341 | 26 | 2331 |
| **Fixed model (95% CI)** | 2.47 [2.04, 3.00] | | | | **Fixed model (95% CI)** | 3.02 [1.94, 4.72] | | | |
| **Heterogeneity** | Chi² = 4.22, df = 5 (P = 0.52); I² = 0% | | | | **Heterogeneity** | Chi² = 7.84, df = 5 (P = 0.17); I² = 36% | | | |
| **Test for overall effect** | Z = 9.17 (P < 0.00001) | | | | **Test for overall effect** | Z = 4.86 (P < 0.00001) | | | |
