# Supplementary figures and images for: Safety and Efficacy of Treatment with/without Ramucirumab in Advanced or Metastatic Cancer: A Meta-Analysis of 11 Global, Double-Blind, Phase 3 Randomized Controlled Trials
Source: J Oncol. 2022 Nov 21;2022:2476469. doi: 10.1155/2022/2476469 (PMC9705087; doi:10.1155/2022/2476469)

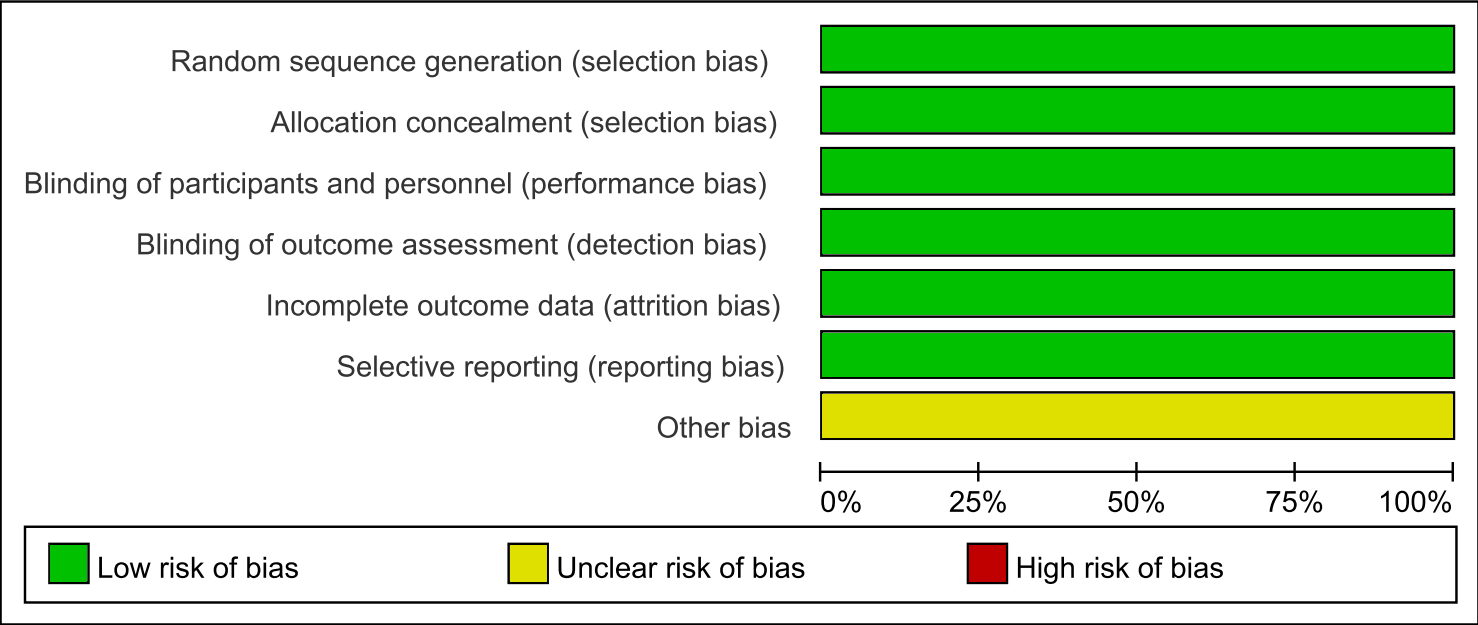

Supplement: Supplementary Materials — Table S1: PubMed Search Strategy of studies. Table S2: Geographic region in the Intent-to-Treat Population of Phase 3 RCTs. Table S3: Detailed data for TEAEs of special interest. Table S4: Detailed data for TEAEs. Figure S1. Risk of bias graph: judgements about each risk of bias item presented as percentages across all included studies. Figure S2. Risk of bias summary: judgements about each risk of bias item for each included study. [file 2476469.f1.zip › Figure S1.pdf]
